# Supplementary material for: Orphan G protein-coupled receptor GPRC5B controls macrophage function by facilitating prostaglandin E receptor 2 signaling
Source: Nat Commun. 2025 Feb 7;16:1448. doi: 10.1038/s41467-025-56713-0 (PMC11805951; doi:10.1038/s41467-025-56713-0)
Supplement: Supplementary file 1 — Supplementary Information [file 41467_2025_56713_MOESM1_ESM.pdf]

Supplemental Information for Kwon *et al.*, 2024:

Orphan G-protein-coupled receptor GPRC5B controls macrophage function by facilitating prostaglandin E receptor 2 signaling

This pdf includes:

1. Supplemental Figures
2. Supplemental Tables
3. Uncropped Blots
4. FACS gating strategies

1. Supplemental Figures for Kwon et al., 2024

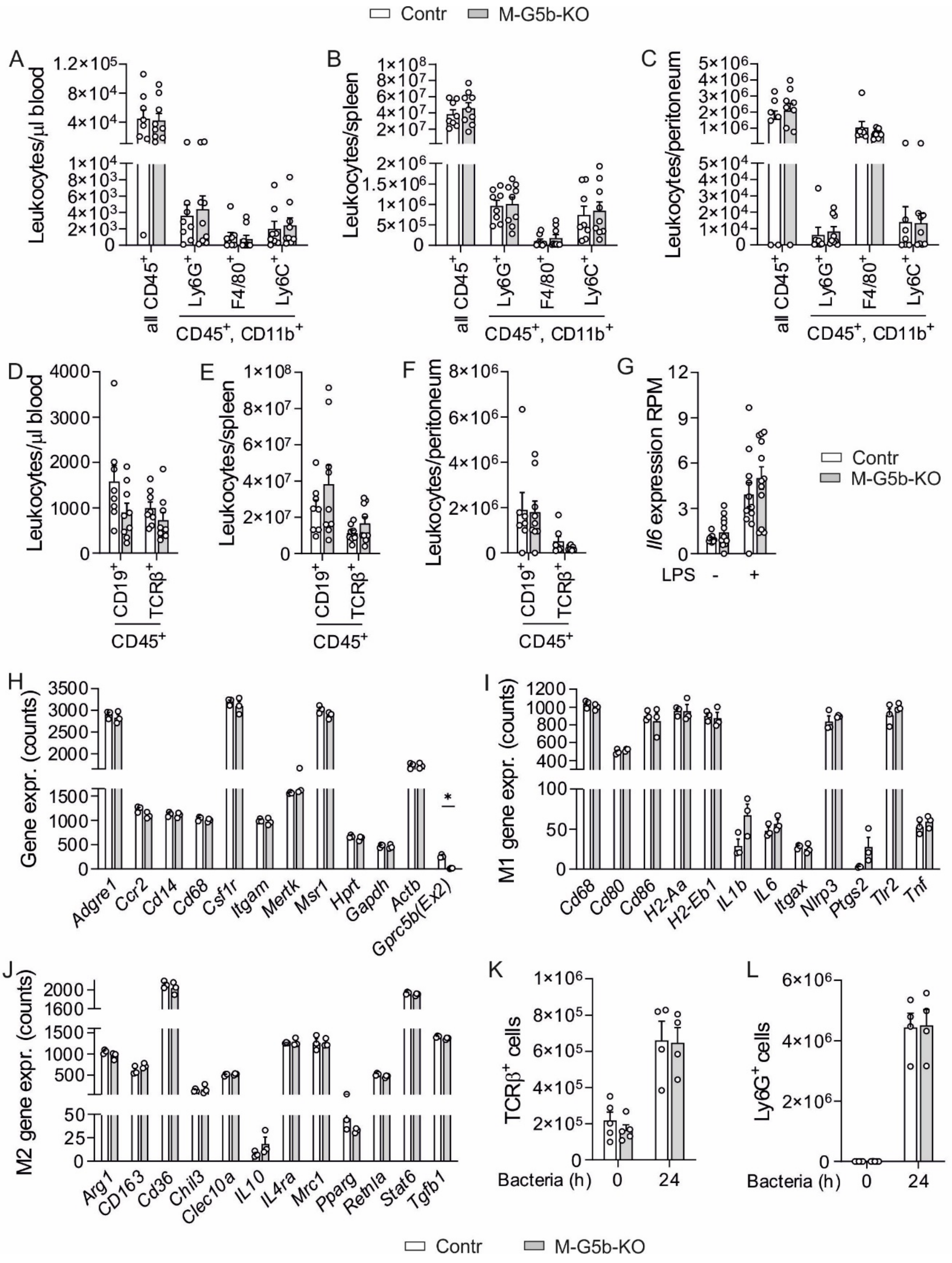

Supplemental Figure 1: A-C, Total numbers of leukocytes (CD45$^+$), neutrophils (CD45$^+$, CD11b$^+$, Ly6G$^+$, macrophages (CD45$^+$, CD11b$^+$, F4/80$^+$, Ly6G$^-$, Ly6C$^-$,) and monocytes (CD45$^+$, CD11b$^+$, Ly6C$^+$, Ly6G$^-$, F4/80$^-$) in blood (A), spleen (B), and peritoneal cavity (C) (7-9 mice per group). **D-F,** Total numbers of B cells (CD45$^+$, CD19$^+$) and T cells (CD45$^+$, TCRβ$^+$, Ly6G$^-$, CD11b$^-$) in blood (D), spleen (E), and peritoneal

cavity (F) (n=7-9). **G,** *Il6* expression in RPM was determined by qRT-PCR under basal conditions and after 6 h of stimulation with LPS 1 μg/ml (n=10-15, data normalized to *Gapdh* and control set to 1). **H-J,** Library size-normalized counts detected by RNA sequencing in RPM isolated from murine peritoneal lavage fluid (3 mice per group). **K,L,** Numbers of TCRβ$^+$ T cells (K) or Ly6G$^+$ neutrophilic granulocytes (L) before and 24 h after i.p. injection of fecal bacteria (n=4-5).

Data are means ± SEM; comparisons between genotypes were performed using two-way ANOVA with Sidak's multiple comparisons test (A-G,K,L) or two-sided Mann-Whitney test with the two-stage step-up method of Benjamini, Krieger and Yekutieli (H-J). n, number of individual mice. Source data are provided as a Source Data file.

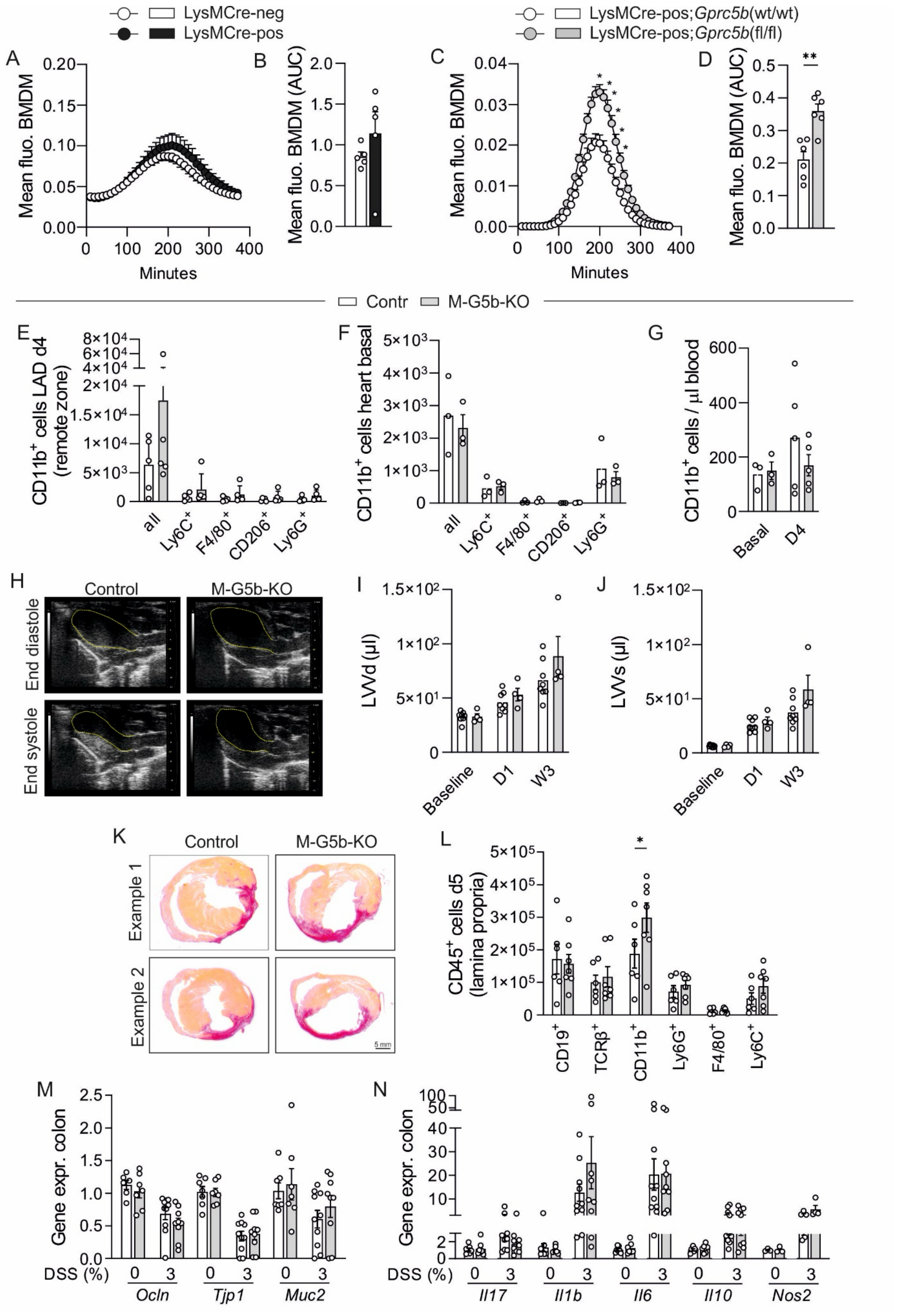

LysMCre-neg
LysMCre-pos
LysMCre-pos;Gprc5b(wt/wt)
LysMCre-pos;Gprc5b(fl/fl)
A
B
C
D
Mean fluo. BMDM
Mean fluo. BMDM (AUC)
Minutes
**
Contr
M-G5b-KO
E
F
G
CD11b+ cells LAD d4 (remote zone)
CD11b+ cells heart basal
CD11b+ cells / µl blood
all
Ly6C+
F4/80+
CD206+
Ly6G+
Basal
D4
H
Control
M-G5b-KO
End diastole
End systole
I
J
LVVd (µl)
LVVs (µl)
Baseline
D1
W3
K
Control
M-G5b-KO
Example 1
Example 2
5 mm
L
CD45+ cells d5 (lamina propria)
CD19+
TCRβ+
CD11b+
Ly6G+
F4/80+
Ly6C+
M
N
Gene expr. colon
DSS (%)
Ocln
Tjp1
Muc2
Il17
Il1b
Il6
Il10
Nos2

Supplemental Figure 2: A-D, Comparison of different control groups: Phagocytic activity of M0 BMDM derived from the indicated mouse lines was determined by uptake of pHrodo E. coli bioparticles: A+C: exemplary curves; B+D: statistical evaluation of areas under curve (AUC) (n=5-6 per group). **E-K, Myocardial infarction model: E-G,** Flow cytometric analysis of CD11b-positive cells in the remote zone of hearts harvested 4 days after infarction (E, n=5), in healthy hearts (F, n=3), or in the peripheral blood before (n=3) and 4 days after infarction (G, n=5). **H-J,** Echocardiographic analysis of left ventricular volumes in diastole (LVVd, I) and systole (LVVs, J) before and after infarction (8 controls, 4 KOs): (H) representative images of the cardiac long axis in B-mode, hatched lines indicate the volume of the left ventricle; (I,J) statistical evaluation of cardiac parameters calculated from analyses shown in H. **K,** Representative picrosirius red-stained sections underlying scar size analyses shown in main Fig. 2K. **L-N, DSS colitis**: **L**, Flow cytometric analysis of immune cell infiltration into the colonic lamina propria at day 5 after initiation of DSS treatment. **M,N,** Expression of genes encoding tight-junction protein occluding (*Ocln*) and ZO-1 (*Tjp1*) or mucin 2 (*Muc2*) (M) or different inflammatory markers (N) on day 6 of DSS treatment (n=7-10; same mice as in main Figure 2).

Data are means ± SEM; comparisons between genotypes were performed using two-way repeated measures ANOVA with Sidak's multiple comparisons test (A,C,I,J), unpaired two-sided t test (B,D), two-way ANOVA with Sidak's post hoc test (E-G,L-N). **, $P<0.01$; *, $P<0.05$; n, number of individual mice. Source data are provided as a Source Data file.

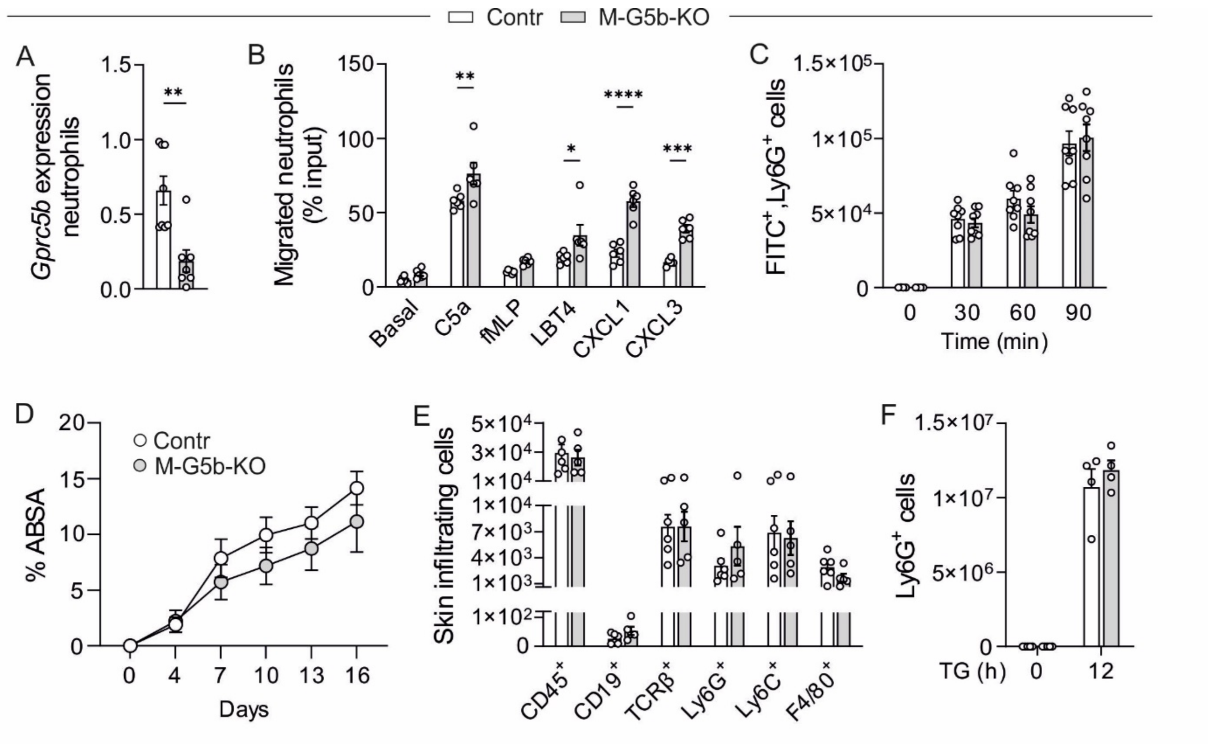

Supplemental Figure 3: Phenotypes in GPRC5B-deficient neutrophils A, Knockout efficiency was determined by qRT-PCR in Ly6G-positive cells harvested from bone marrow (n=8, data normalized to *Gapdh* and controls set to 1). **B,** Basal and chemoattractant-induced migration was induced in Ly6G-positive neutrophils isolated from murine bone marrow in the transwell system (C5a: 20 ng/ml; fMLP: 10 nM, LTB4: 1 uM, CXCL1: 1 uM, CXCL3: 1 uM; n=6). **C,** Phagocytic activity of Ly6G-positive neutrophils isolated from murine bone marrow was determined by uptake of pHrodo E. coli bioparticles (n=8). **D,E,** EBA in control and M-G5b-KO mice: Affected body surface area (ABSA, D) and immune cell infiltration into skin biopsies obtained on d16 (E) (n=5). **F,** The number of Ly6G-positive neutrophils was determined in control and M-G5b-KOs in the basal state and 12 h after i.p. administration of 1 ml Brewer's thioglycolate (TG) solution (n=4).

Data are means ± SEM; comparisons between genotypes were performed using two-sided Mann-Whitney test (A), two-way ANOVA (B,C,E,F) or two-way RM-ANOVA (D) with Sidak' correction for multiple testing. ****, $P<0.0001$; ***, $P<0.001$; **, $P<0.01$; n, number of individual mice. Source data are provided as a Source Data file.

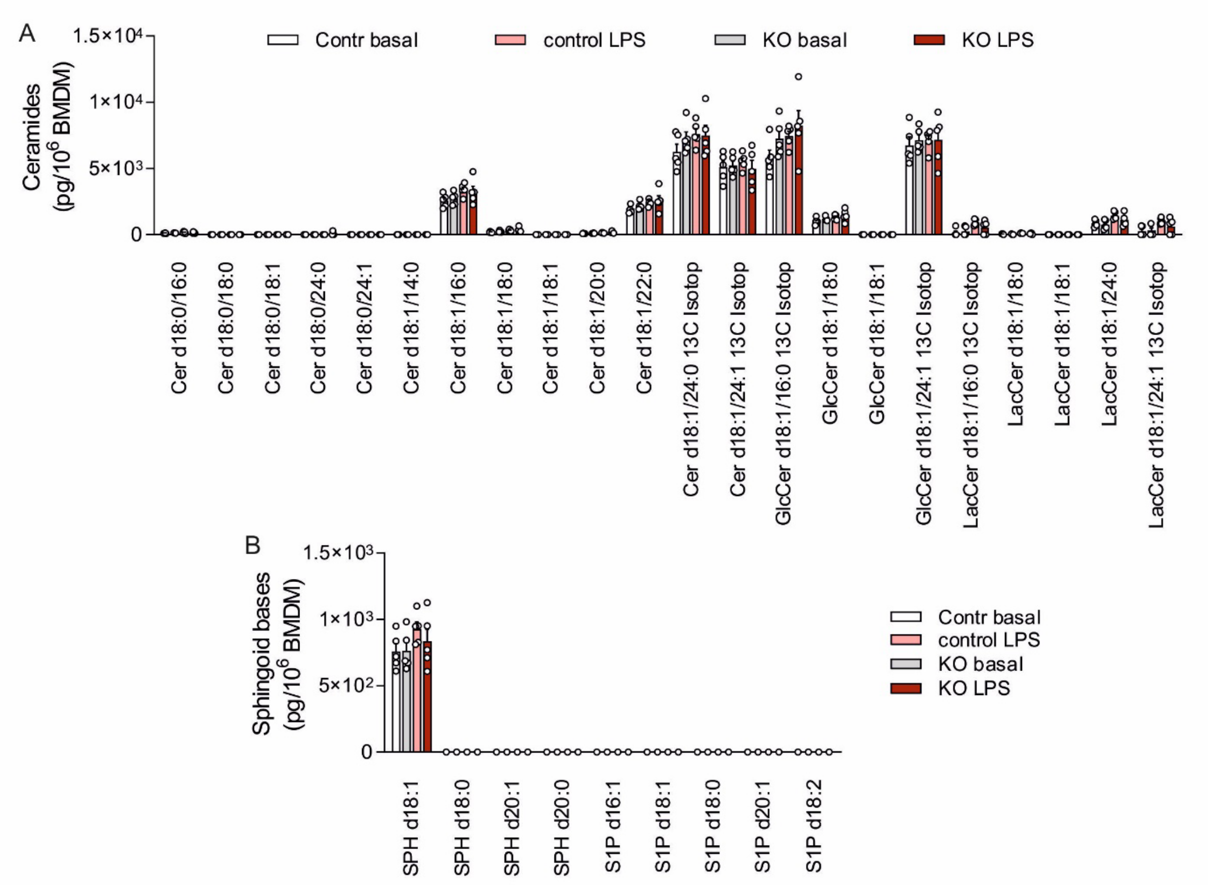

Supplemental Figure 4: Levels of ceramides (A) and sphingoid bases (B) were determined by LC-MS/MS in 2.5x10^{5} resting and LPS-treated (1 µg/ml, 6h) M0 BMDM cells (n=3). For highly abundant ceramides, the 13C Isotope was analysed to adjust the sensitivity.

Data are means ± SEM; differences were analyzed using Kruskal-Wallis test with Dunn's correction for multiple testing. Source data are provided as a Source Data file.

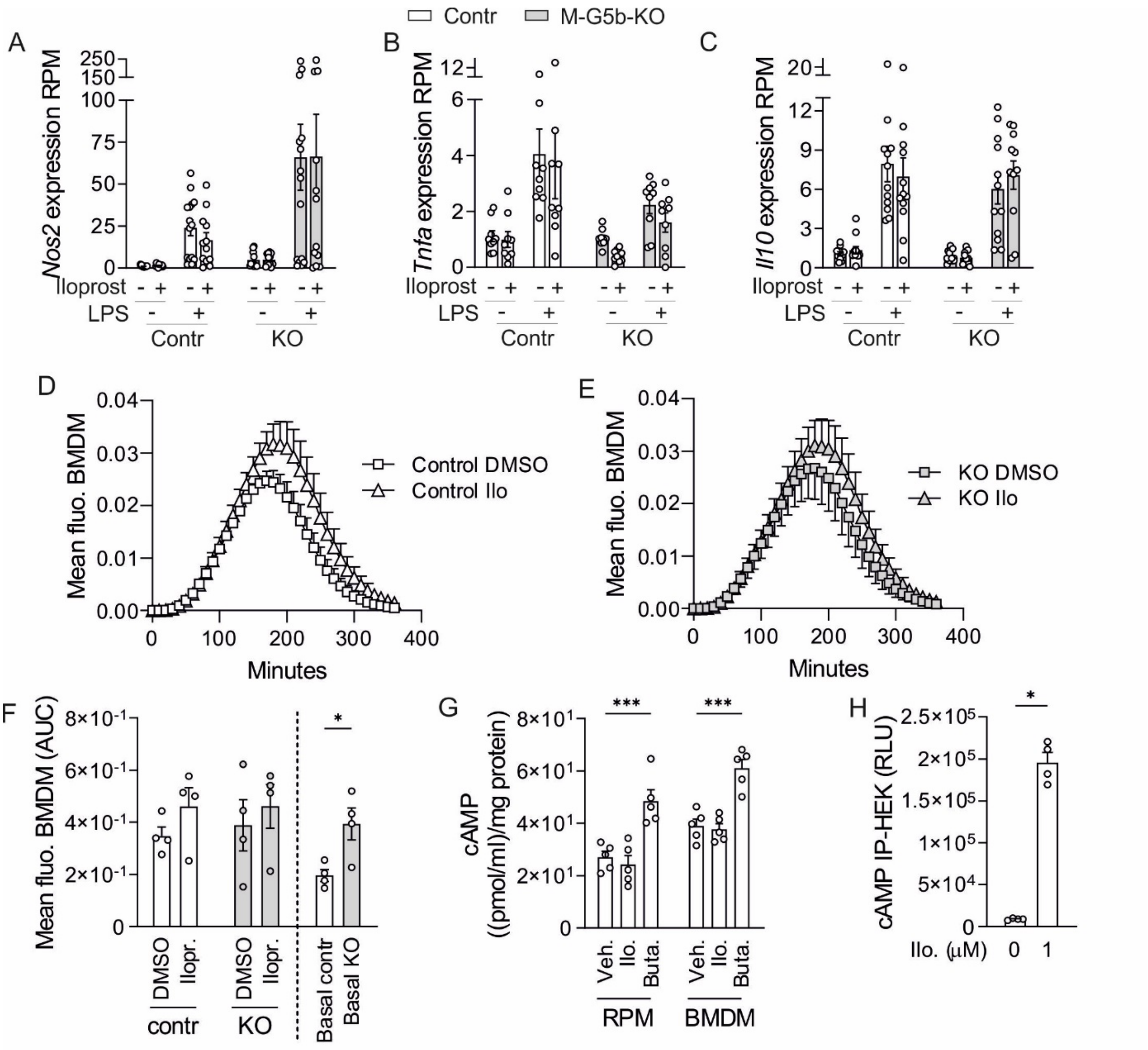

Supplemental Figure 5: A-C, Effect of iloprost (1 μM) on inflammatory gene expression in RPM under basal conditions and after 6 h of 1 μg/ml LPS stimulation (n=12-15, data normalized to GAPDH and basal control set to 1). **D,E,** Effect of iloprost (1 μM) on phagocytosis of pHrodo E. coli bioparticles in control (D) and KO (E) RPM (n= 5). **F,** Statistical evaluation of AUC for D and E (note: DMSO increases basal phagocytosis in control cells, but not in KO; basal hyperphagocytosis of KO cells (see right side of graph) is therefore less prominent in the presence of DMSO). **G,** cAMP production induced by iloprost (Ilo.) or EP2 agonist butaprost (Buta.) in RPM and M0 BMDM (n=5). **H,** cAMP production in response to stimulation with Iloprost (Ilo.) in HEK cells transfected with cAMP GloSensor plasmid and plasmids encoding the IP receptor (n=4).

Data are means ± SEM; differences between genotypes (A-C) or between vehicle- and agonist-treated groups (F-H) were analyzed using two-way ANOVA and Sidak's post hoc test (A-C) or Kruskal-Wallis test with Dunn's correction for multiple testing (F), one-way ANOVA with Tukey's post hoc test (G), or two-sided Mann-Whitney test (H). ***, $P < 0.001$; *, $P < 0.05$; n, number of experiments or mice per group; veh., vehicle. Source data are provided as a Source Data file.

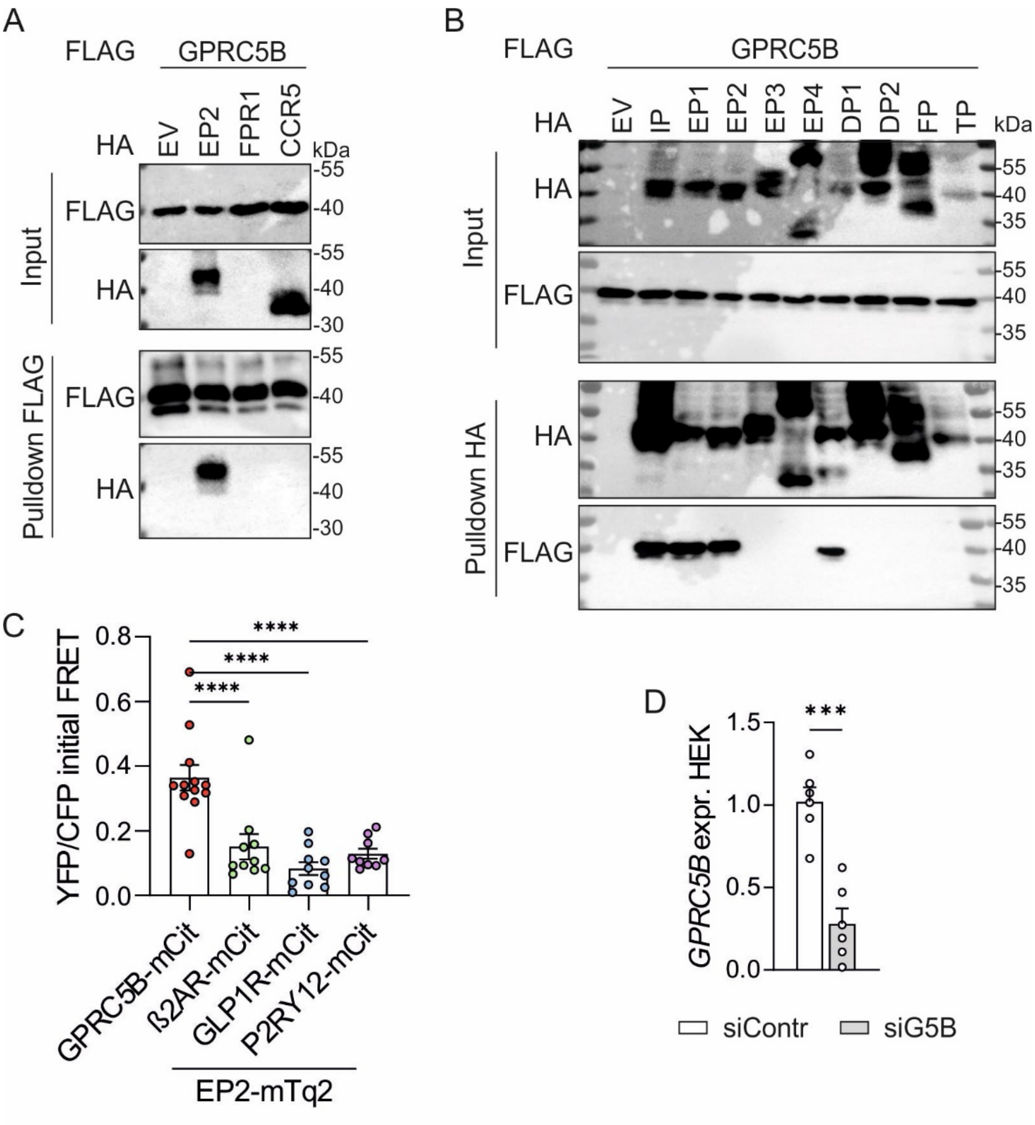

Supplemental Figure 6: A,B, Western blot detection of HA and FLAG signals in lysates of HEK cells expressing GPRC5B-FLAG/Myc in combination with different HA-tagged receptors before ("input") and after immunoprecipitation of FLAG-tagged receptors ("Pulldown FLAG" in A) or immunoprecipitation of HA-tagged receptors ("Pulldown HA" in B). Receptor FPR1 failed to be expressed in A. **C,** Elevated Förster resonance energy transfer (FRET) between EP2 and GPRC5B compared to randomly chosen other GPCRs: HEK cells were transfected with EP2-mTurqouise2 (EP2-mTq2) and different mCitrine (mCit)-labeled receptors as indicated and initial FRET was determined (n=9-12 individual cells in 2-3 independent experiments). **D,** GPRC5B expression in HEK cells after transfection with control siRNA (siContr) or siRNA directed against GPRC5B (siG5B) was determined by qRT-PCR (n=6).

Data are means ± SEM; differences between genotypes were analyzed using one-way ANOVA with Tukey's multiple comparison test (C) or unpaired two-sided t test (D). ***, *P* < 0.001; ****, *P* < 0.0001; EV, empty vector; n, number of independent experiments. Source data are provided as a Source Data file.

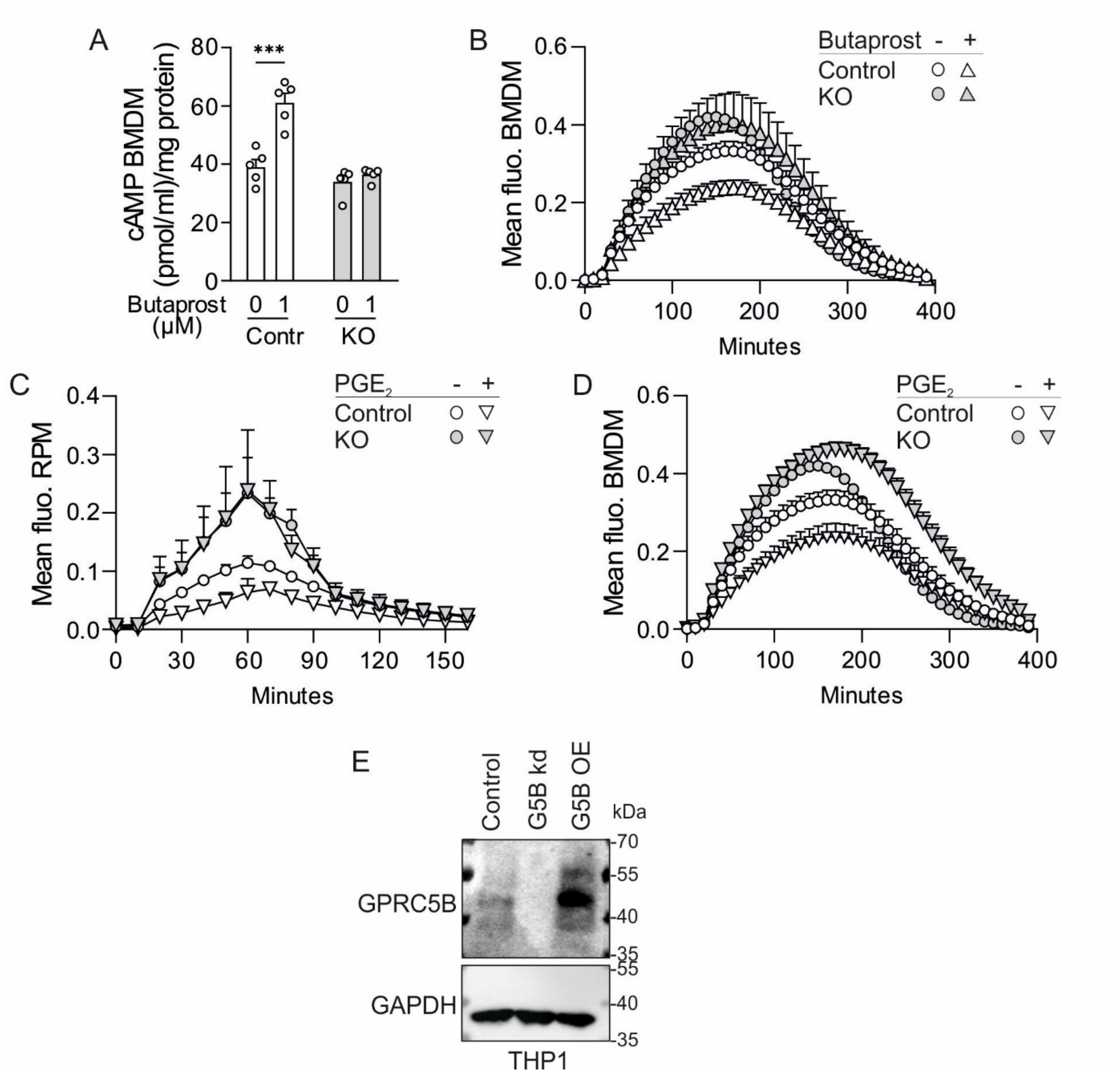

Supplemental Figure 7: A, Butaprost-induced cAMP production was determined by ELISA in M0 BMDM (n=5). **B-D,** The effect of butaprost (B) or PGE_2 (C,D) on uptake of pHrodo E. coli bioparticles phagocytic was determined in M0 BMDM (B,D) and RPM (C) (n=4; untreated cells receive vehicle DMSO only). **E,** The effect of siRNA-mediated GPRC5B knockdown (G5B kd) or GPRC5B overexpression (G5B OE) was determined in THP1 by immunoblotting (Control: EV + control siRNA; G5B kd: EV + GPRC5B siRNA; G5B OE: GPRC5B expression plasmid + control siRNA; GAPDH as loading control).

Data are means ± SEM; comparisons between vehicle- and agonist-treated groups were performed using unpaired two-sided t test (A). ****, $P < 0.0001$; **, $P < 0.01$; *, $P < 0.05$; n, number of independent experiments or mice per group. Source data are provided as a Source Data file.

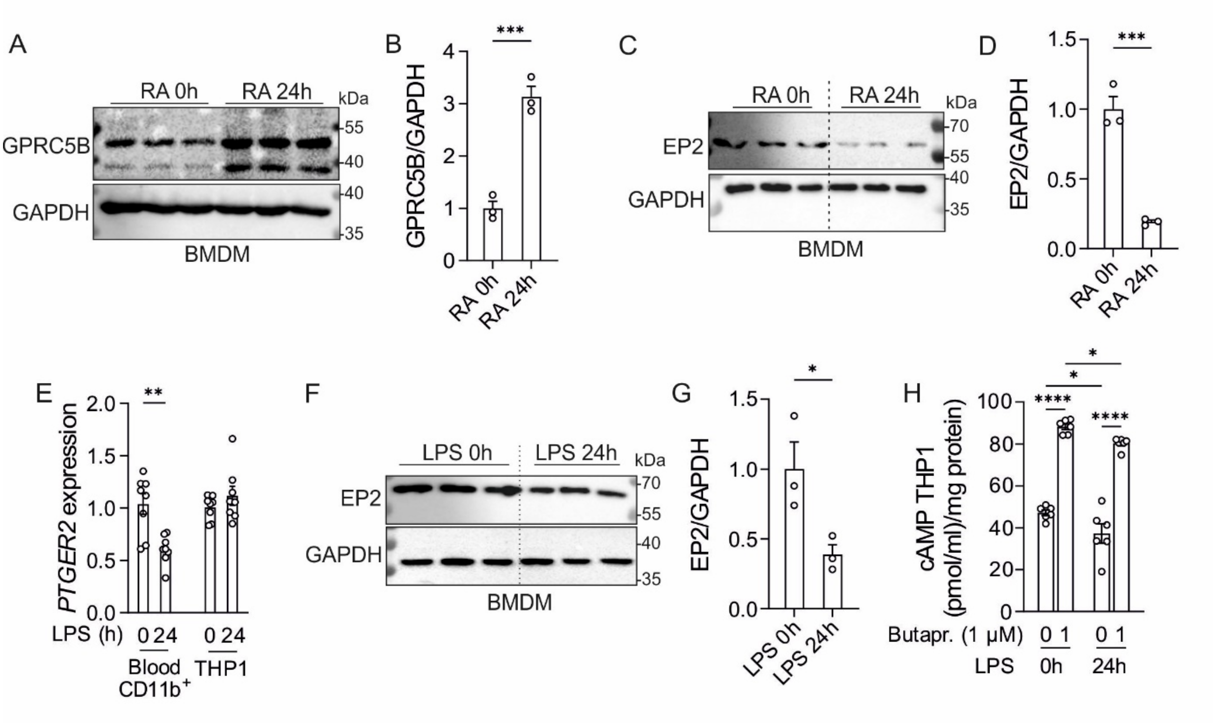

Supplemental Figure 8: A,B, Effect of RA (1 µM, 24h) on GPRC5B expression in BMDM was determined by immunoblotting: representative immunoblot (A) and densitometric analysis of signal intensity (B), GAPDH as loading control (n=3). **C,D,** Effect of RA (1 µM, 24h) on EP2 expression in BMDM was determined by immunoblotting: representative immunoblot (C) and densitometric analysis of signal intensity (D), GAPDH as loading control (n=3). **E,** *PTGER2* expression was determined by qRT-PCR in human blood CD11b-positive cells and THP1 cells after 24 h exposure to 1 µg/ml LPS (n=8; data normalized to *GAPDH* and average basal set to 1). **F,G,** Effect of LPS (1 µg/ml, 24h) on EP2 expression in BMDM was determined by immunoblotting: representative immunoblot (F) and densitometric analysis of signal intensity (G), GAPDH as loading control (n=3). **H,** Butaprost-induced cAMP production in M0 BMDM exposed to LPS or vehicle for 24 h (n=6; data normalized to *Gapdh* and average basal set to 1).

Data are means ± SEM; comparisons between groups were performed using unpaired two-sided t test (B,D,E,G) or two-way ANOVA with Sidak's post hoc test (H). ****, $P < 0.0001$; **, $P < 0.01$; *, $P < 0.05$; n, number of independent experiments or mice per group. Source data are provided as a Source Data file.

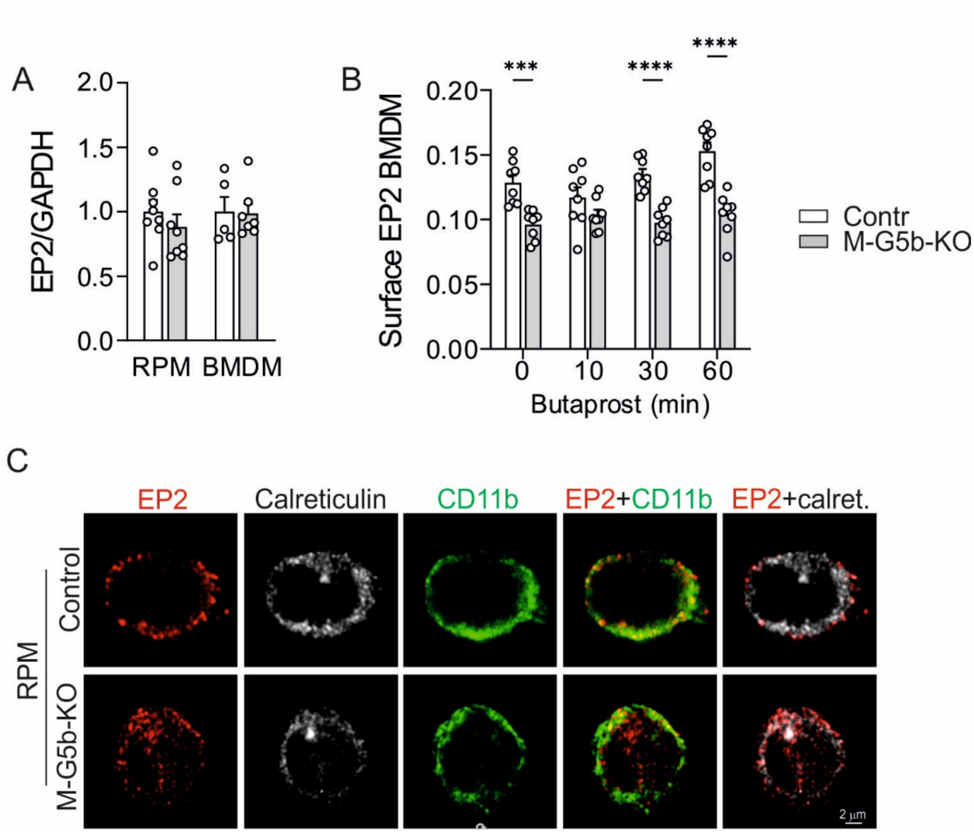

Supplemental Figure 9: A, Quantification of EP2 signal intensity relative to GAPDH in immunoblots of RPM and BMDM lysates (n=5-8). **B,** ELISA-based detection of EP2(ec) in the plasma membrane of M0 BMDM after different times of stimulation with 1 µM butaprost (n=8). **C,** RPM from control and KO mice were fixed, permeabilized, and stained for EP2(ec) and ER marker calreticulin, and then stained with anti-CD11b antibodies (for plasma membrane). Shown are exemplary photomicrographs, statistical evaluation is in Fig. 5K (n=10).

Data are means ± SEM; comparisons between genotypes were performed using unpaired two-sided t test (A) or two-way ANOVA with Sidak's post hoc test (B). N, number of independent samples. ****, $P < 0.0001$; ***, $P < 0.001$; **, $P < 0.01$; *, $P < 0.05$. Source data are provided as a Source Data file.

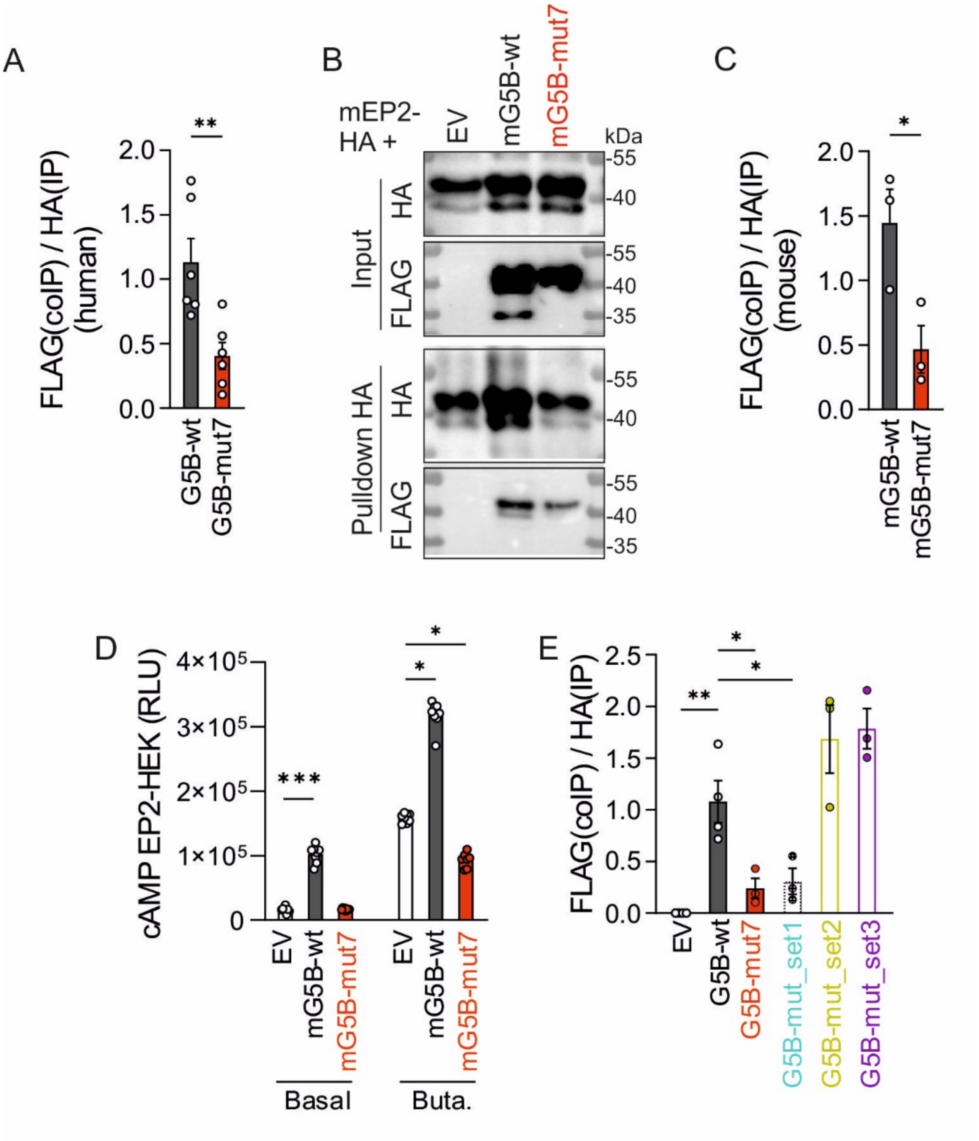

Supplemental Figure 10: A, Densitometric analysis of signal strength for co-immunoprecipitated human GPRC5B-FLAG (FLAG(coIP)) relative to immunoprecipitated human HA-EP2 (HA(IP)) in HEK cells expressing wildtype GPRC5B (G5B-wt) or GPRC5B-mut7 (G5B-mut7) (n=6). **B,** Western blot detection of HA and FLAG signals in lysates of HEK cells expressing HA-tagged murine EP2 (mEP2-HA) in combination with empty vector (EV), FLAG-tagged murine G5B-wt or murine G5B-mut7: "input" shows lysates before, "pulldown HA" after immunoprecipitation with anti-HA beads. **C,** Densitometric analysis of signal strength for co-immunoprecipitated murine GPRC5B-FLAG (FLAG(coIP)) relative to immunoprecipitated murine HA-EP2 (HA(IP)) in HEK cells expressing murine wildtype GPRC5B (mG5B-wt) or murine GPRC5B-mut7 (mG5B-mut7) (n=3). **D,** Butaprost (1 µM)-induced cAMP production in HEK cells transfected with cAMP GloSensor plasmid, murine EP2-HA, and wild type or mutant murine GPRC5B as indicated (n=8). **E,** Quantification of the effect of different GPRC5B mutations on co-immunoprecipitation of GPRC5B-FLAG with HA-EP2 in HEK cells (n=3-4).

Data are means ± SEM; comparisons with empty vector-transfected samples were performed using unpaired two-sided t test (A,C), Kruskal-Wallis test with Dunn's correction for multiple testing (D), or one-way ANOVA with Dunnett's multiple comparison test (E). N, number of independent samples. ****, $P < 0.0001$; ***, $P < 0.001$; **, $P < 0.01$; *, $P < 0.05$. Source data are provided as a Source Data file.

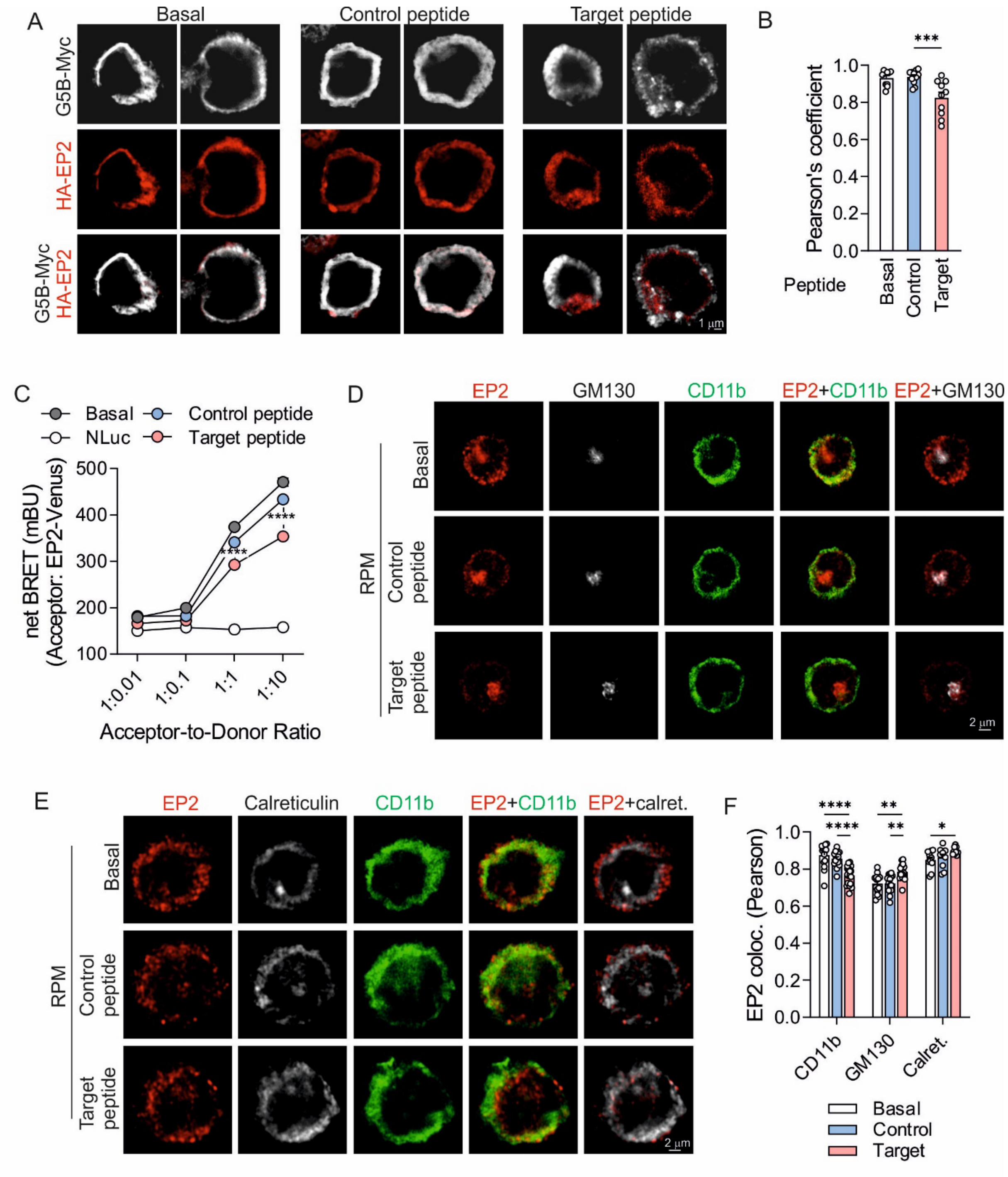

Supplemental Figure 11: A,B, Effect of target and control peptides on the co-localization of GPRC5B-FLAG/Myc with HA-EP2 in HEK cells. 48 h after co-transfection of HA-EP2 and GPRC5B-FLAG/Myc plasmids, HEK cells were cultured for 1 h with our without 10 µM control or target peptide. Cells were then fixed, permeabilized, and stained with anti-Myc and anti-HA antibodies and the colocalization of both signals was determined (A, exemplary photomicrographs, B, statistical evaluation of the Pearson's coefficient, n=10-11 cells). **C,** Bioluminescence resonance energy transfer (BRET) between GPRC5B-NLuc (donor) and EP2-Venus (acceptor) in HEK cells transfected with different ratios of donor and

acceptor plasmids. 48 h after transfection, cells were cultured for 1 h in the presence or absence of 10 µM control or target peptide, then BRET was determined. Data are expressed as ratio of acceptor emission (535 nm) to donor emission (460 nm) (n=4 per condition). **D,E,** Effect of target and control peptides on EP2 localization in RPM: wildtype RPM were treated with solvent (basal), control peptide, or target peptide (1 µM each peptide) for 1 h, then cells were fixed, permeabilized, stained either for EP2(ec) and Golgi marker GM130 (B) or EP2(ec) and ER marker calreticulin (C), and then stained with anti-CD11b antibodies (for plasma membrane). **F,** Statistical evaluation of the colocalization of EP2(ec) with CD11b, GM130 or calreticulin (n=11-18 cells per condition).

Data are means ± SEM; comparisons between treatment groups were performed using one-way ANOVA with Tukey's post hoc test (B) or two-way ANOVA with Sidak's post hoc test (C,F). N, number of independent samples. *, $P < 0.05$; ***, $P < 0.001$; ****, $P < 0.0001$. Source data are provided as a Source Data file.

2. Supplemental Tables for Kwon et al., 2024

Supplemental Table 1: FACS antibodies							
Antibody (anti-)	Isotype	Clone	Species	Fluoro-chrome	Source	Catalog #	Dilution
CCR2 (CD192)	Rat IgG2b, κ	SA203G11	Mouse	APC	Biolegend	150627	1:500
CD11b	Rat IgG2b, κ	M1/70	Mouse, human	BV510	Biolegend	101263	1:500
CD11b	Rat IgG2b, κ	M1/70	Mouse	eFluor450	ebioscience	48-0112-82	1:500
CD19	Rat IgG2a, κ	1D3	Mouse	eFluor450	eBioscience	48-019380	1:500
CD19	rat IgG2a, κ	1D3	Mouse, human	PE	eBioscience	12-0193	1:500
CD206	Rat IgG2b, κ	MR6F3	Mouse	PE	eBioscience	12-2061-82	1:500
CD45	Rat IgG2b, κ	30-F11	Mouse	FITC	BD Biosciences	553079	1:500
F4/80	Rat IgG2a, κ	BM8	Mouse	PE	Biolegend	123110	1:500
F4/80	Rat IgG2a, κ	BM8	Mouse	PE-Cy5	Biolegend	123111	1:500
F4/80	Rat IgG2a, κ	BM8	Mouse	APC-eFluor780	eBioscience	47-4801	1:500
Ly6C	Rat IgG2c, κ	HK1.4	Mouse	PE-Cy7	Biolegend	128017	1:500
Ly6C	Rat IgM, κ	AL-21	Mouse	FITC	BD Biosciences	561085	1:500
Ly6G	Rat IgG2a, κ	1A8	Mouse	BU421	Biolegend	127627	1:500
Ly6G	Rat IgG2a, κ	1A8	Mouse	APC	Biolegend	127613	1:500
MHCII	Rat IgG2b, κ	M5/114.152	Mouse	APC-Cy7	Biolegend	107627	1:500
TCRβ	Armenian Hamster IgG	H57-597	Mouse	BV421	Biolegend	109229	1:500
TCRβ	Armenian Hamster IgG	H57-597	Mouse	PerCp-Cy5,5	Biolegend	109227	1:500
Tim4	Rat IgG2a, κ	RMT4-54	Mouse	PerCp-Cy5.5	Biolegend	130019	1:500
Isotype control	Rat IgG2c, k	315		PE-Cy7	BioLegend	400721	1:500

Supplemental Table 1. Antibodies used for FACS.

Supplemental table 2: Antibodies for immunofluorescence staining						
	**Catalog #**	**Clone**	**Isotype**		**Source**	**Dilution**
HA-tag	ab167171	EPR8030(B)	Rabbit IgG	Monoclonal	Abcam	1:500
c-Myc-tag	sc-40	9E10	Mouse IgG	Monoclonal	Santa cruz	1:100
Calreticulin	PA1-902A		Chicken IgY	Polyclonal	Thermo Fisher Scientific	1:100
GM130	610822	35/GM130	Mouse IgG	Monoclonal	BD Biosciences	1:100
PTGER2 (extracellular)	APR-064		Rabbit IgG	Polyclonal	Alomone Lab	1:500
CD11b	561688	M1/70	Rat IgG	Monoclonal	BD Biosciences	1:500
Chicken IgY, Alexa Fluor 568	A-11041		Goat IgG	Polyclonal	Invitrogen	1:500
Rabbit IgG, Alexa Fluor 594	A-21207		Donkey IgG	Polyclonal	Invitrogen	1:500
Rabbit IgG, Alexa Fluor 647	A-31573		Donkey IgG	Polyclonal	Invitrogen	1:500
Mouse IgG, Alexa Fluor 594	A-11005		Goat IgG	Polyclonal	Invitrogen	1:500
Mouse IgG, Alexa Fluor 647	A-31571		Donkey IgG	Polyclonal	Invitrogen	1:500

Supplemental Table 2. Antibodies used for immunofluorescence staining

3. Uncropped blots used in manuscript for Kwon et al., 2024

Fig 1D

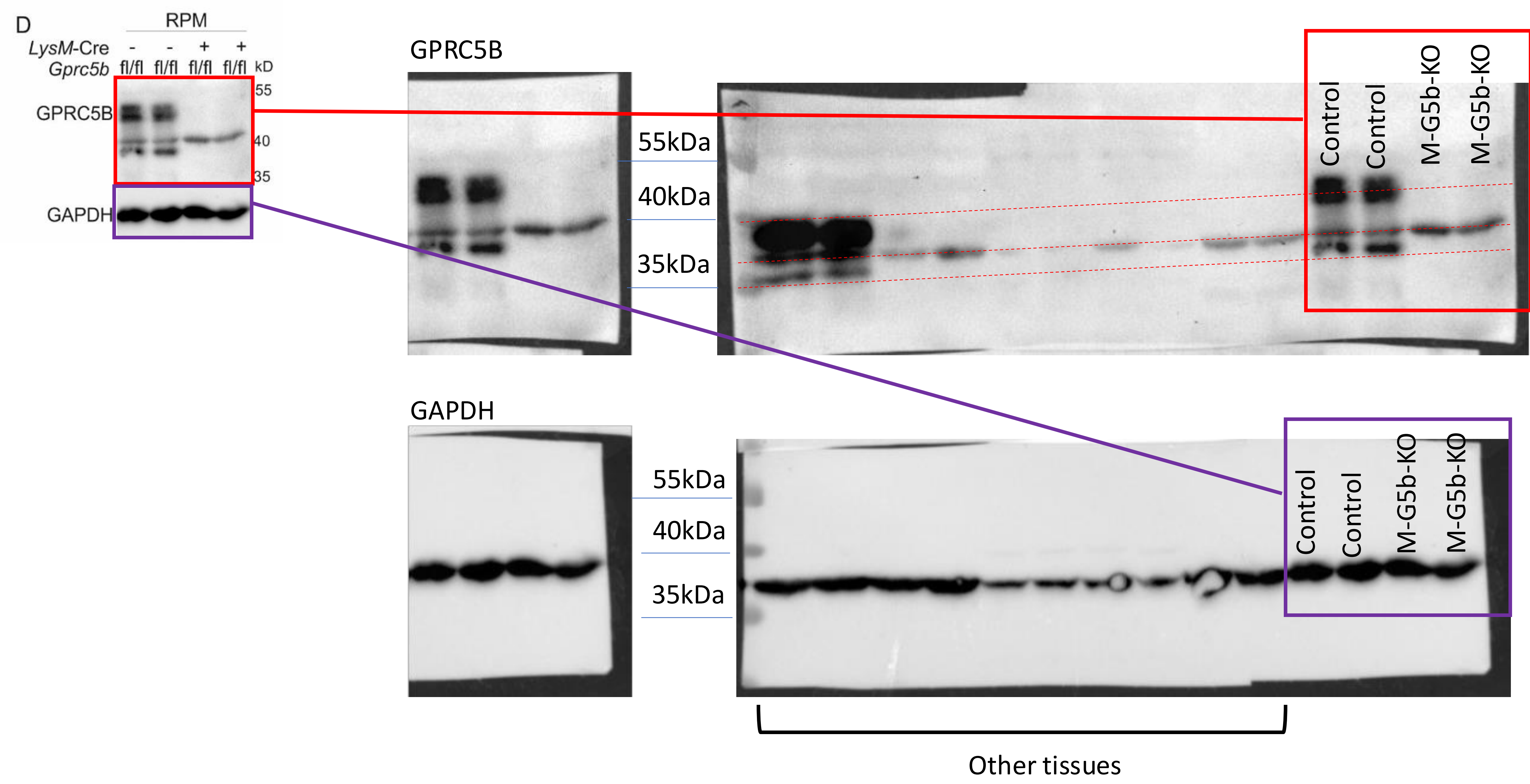
D
RPM
LysM-Cre - - + +
Gprc5b fl/fl fl/fl fl/fl fl/fl kD
GPRC5B
GAPDH
55
40
35
GPRC5B
55kDa
40kDa
35kDa
Control
Control
M-G5b-KO
M-G5b-KO
GAPDH
55kDa
40kDa
35kDa
Control
Control
M-G5b-KO
M-G5b-KO
Other tissues

Fig 3A

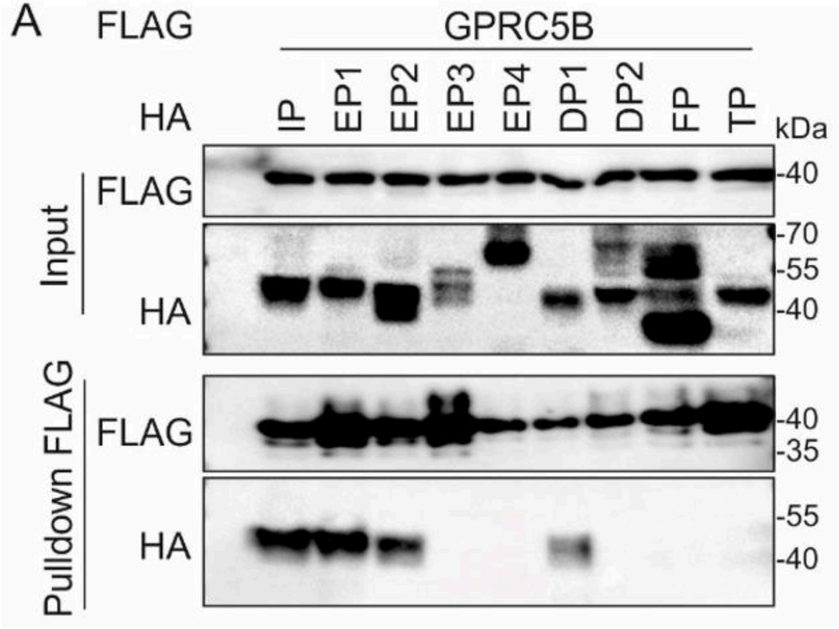
A
FLAG
GPRC5B
HA
IP
EP1
EP2
EP3
EP4
DP1
DP2
FP
TP
kDa
Input
FLAG
HA
Pulldown FLAG
FLAG
HA
-40
-70
-55
-40
-40
-35
-55
-40

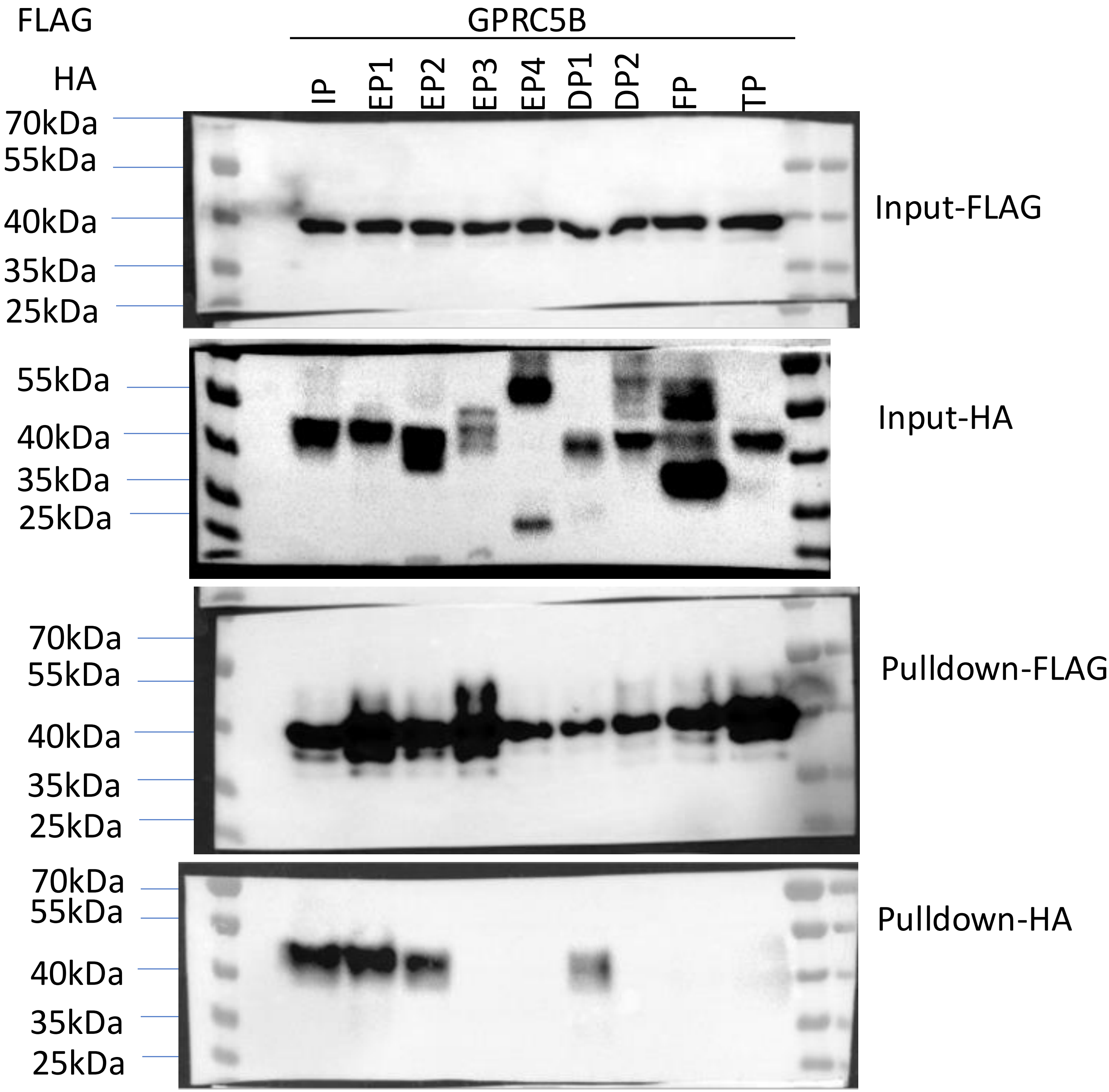
FLAG
GPRC5B
HA
IP
EP1
EP2
EP3
EP4
DP1
DP2
FP
TP
70kDa
55kDa
40kDa
35kDa
25kDa
Input-FLAG
55kDa
40kDa
35kDa
25kDa
Input-HA
70kDa
55kDa
40kDa
35kDa
25kDa
Pulldown-FLAG
70kDa
55kDa
40kDa
35kDa
25kDa
Pulldown-HA

Fig 4A

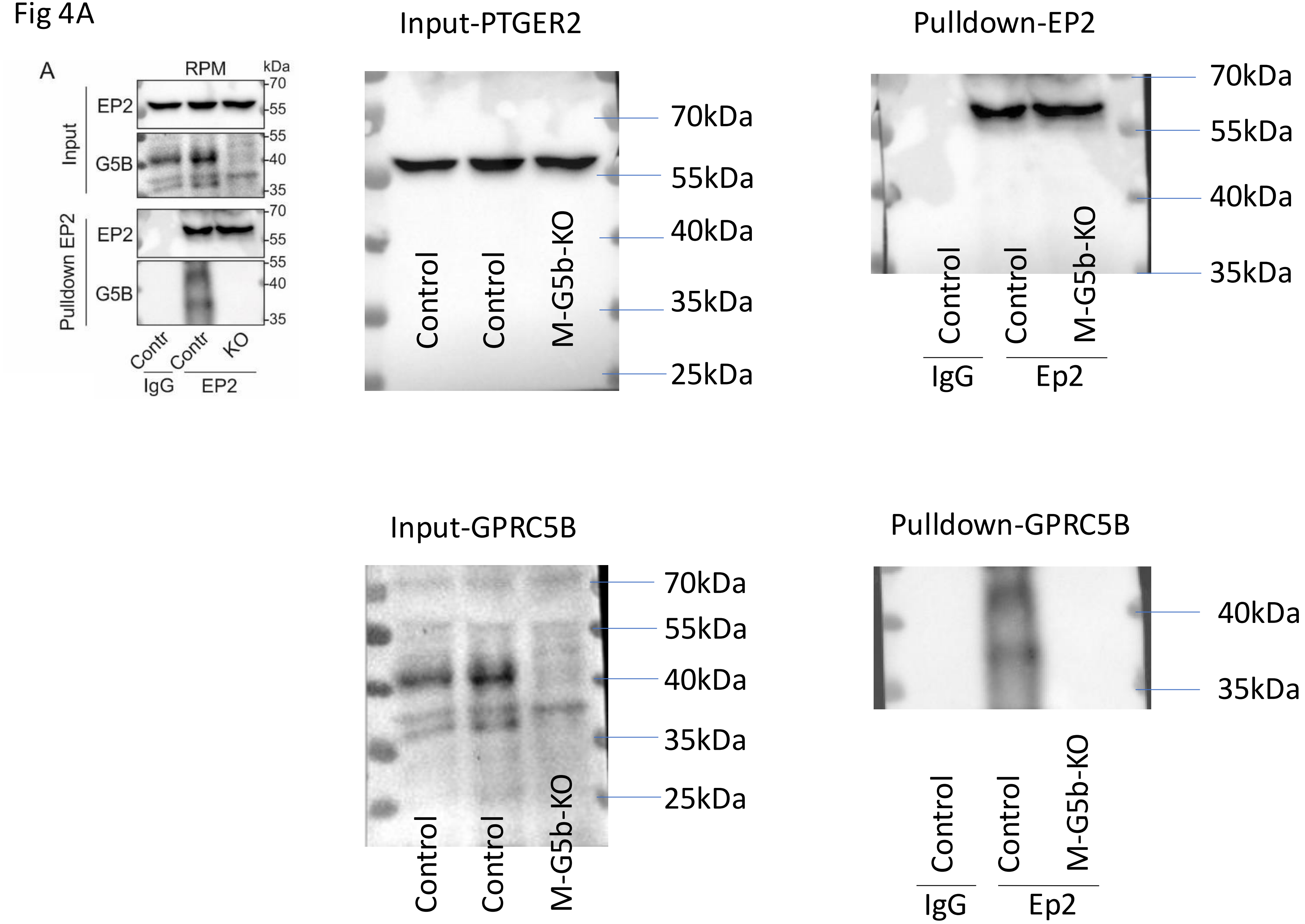
A
RPM
kDa
EP2
G5B
Input
Pulldown EP2
Contr
Contr
KO
IgG
EP2
Input-PTGER2
70kDa
55kDa
40kDa
35kDa
25kDa
Control
Control
M-G5b-KO
Pulldown-EP2
IgG
Ep2
Input-GPRC5B
Pulldown-GPRC5B

Fig 4O

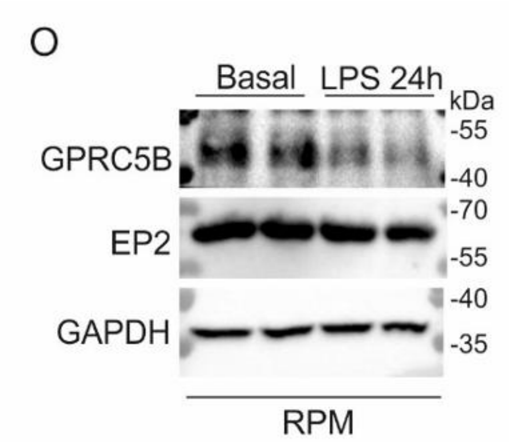
O
Basal
LPS 24h
kDa
GPRC5B
-55
-40
EP2
-70
-55
GAPDH
-40
-35
RPM

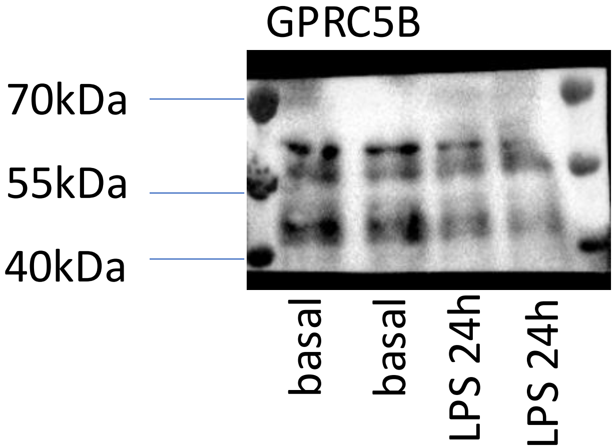
GPRC5B
70kDa
55kDa
40kDa
basal
basal
LPS 24h
LPS 24h

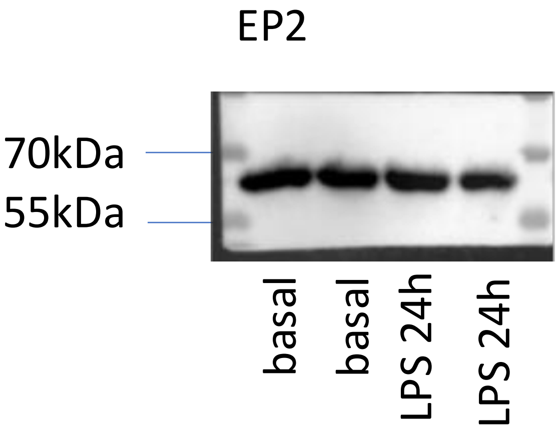
EP2
70kDa
55kDa
basal
basal
LPS 24h
LPS 24h

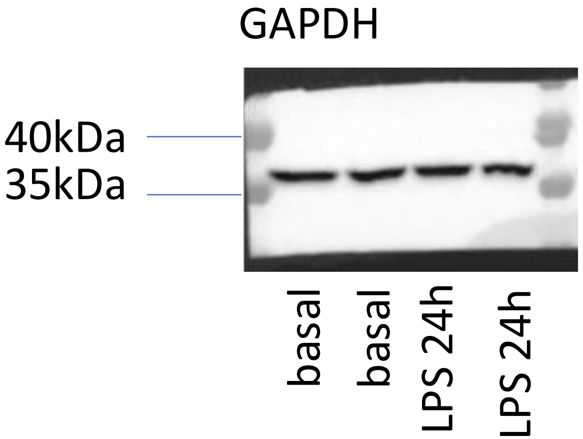
GAPDH
40kDa
35kDa
basal
basal
LPS 24h
LPS 24h

Fig 5C

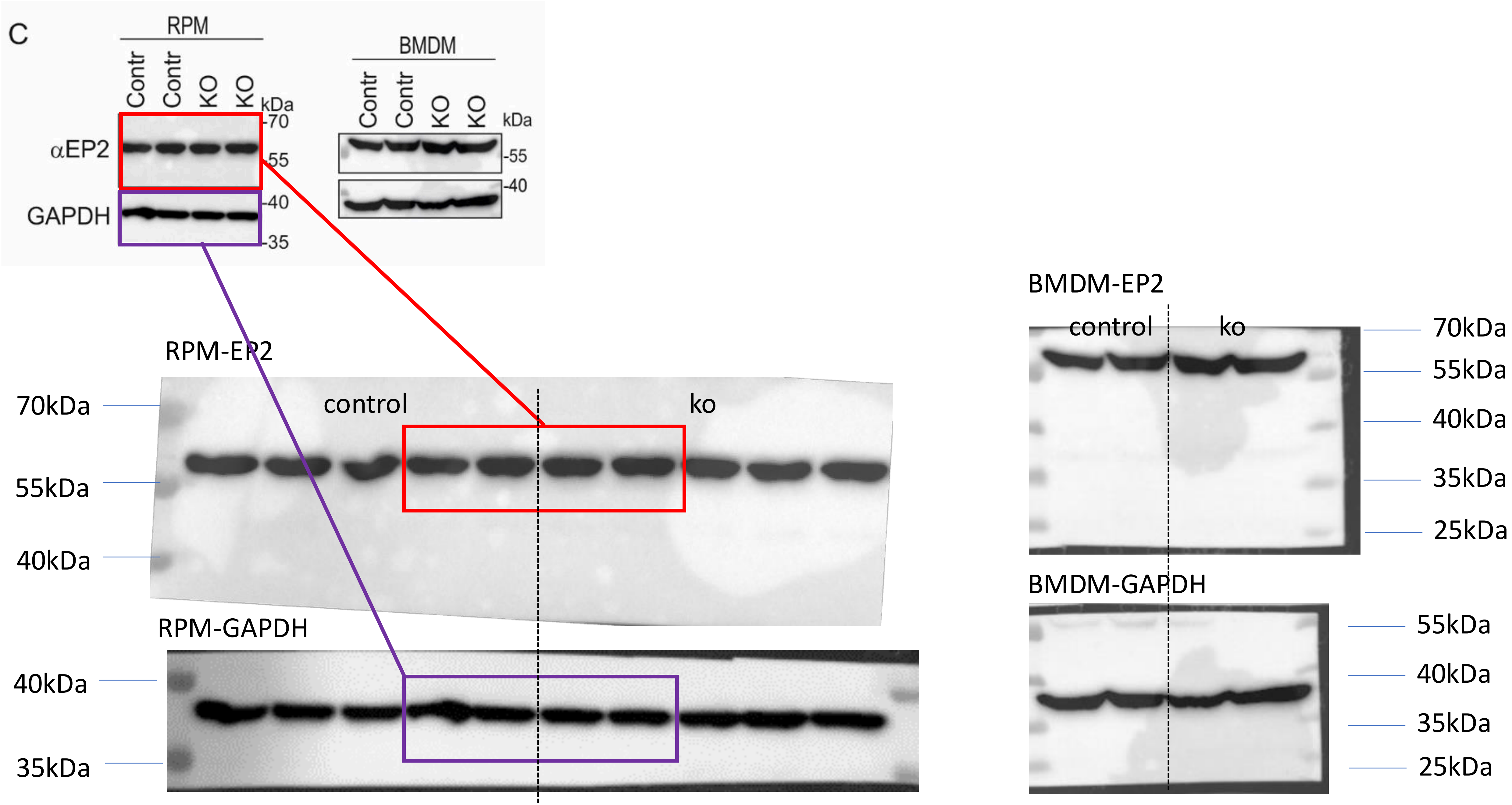
C
RPM
Contr
Contr
KO
KO
kDa
αEP2
GAPDH
70
55
40
35
BMDM
Contr
Contr
KO
KO
kDa
55
40
RPM-EP2
control
ko
70kDa
55kDa
40kDa
RPM-GAPDH
40kDa
35kDa
BMDM-EP2
control
ko
70kDa
55kDa
40kDa
35kDa
25kDa
BMDM-GAPDH
55kDa
40kDa
35kDa
25kDa

Fig 6D

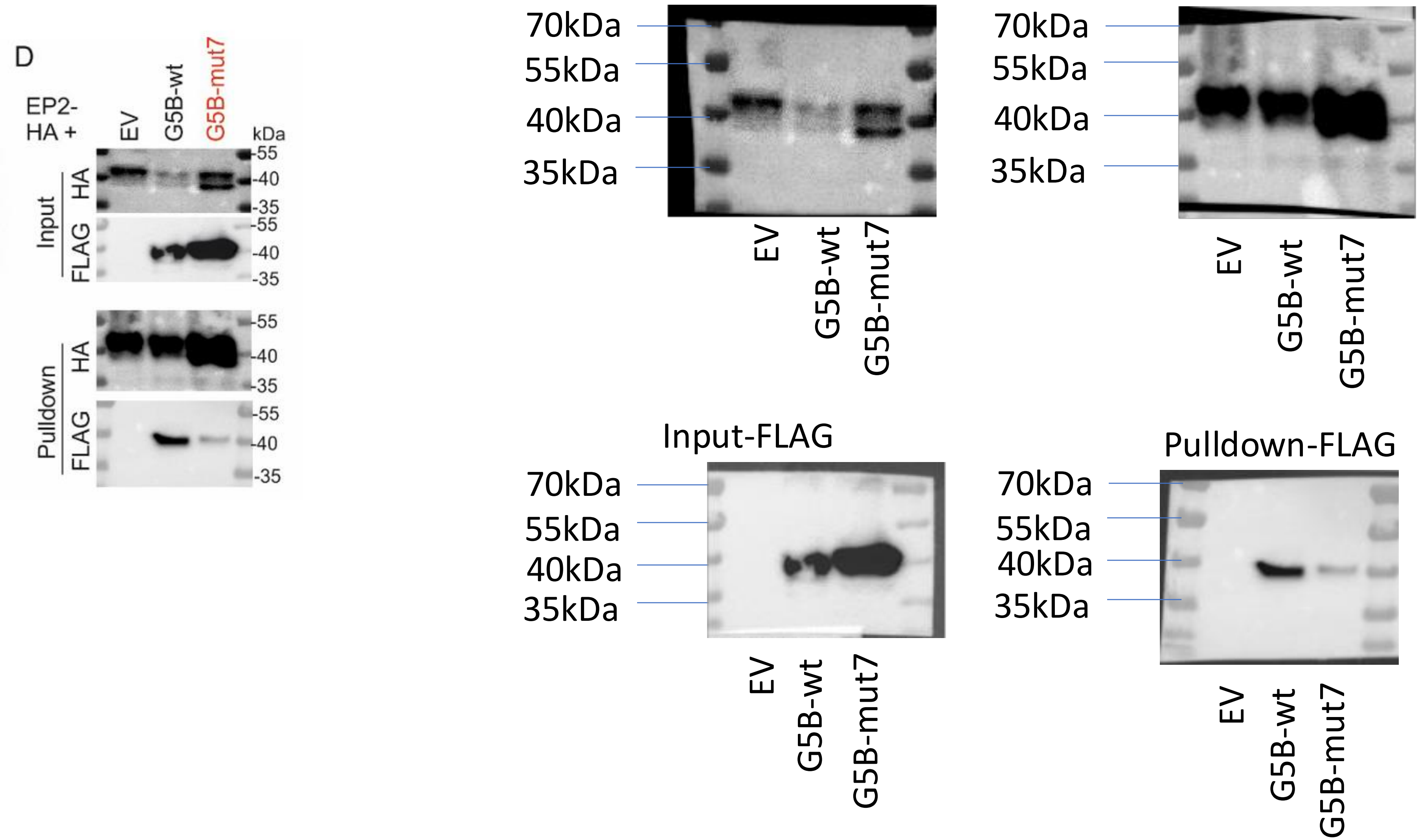

D
EP2-HA +
EV
G5B-wt
G5B-mut7
kDa
55
40
35
Input
HA
FLAG
Pulldown
Input-HA
Pulldown-HA
Input-FLAG
Pulldown-FLAG
70kDa
55kDa
40kDa
35kDa

Fig 6F

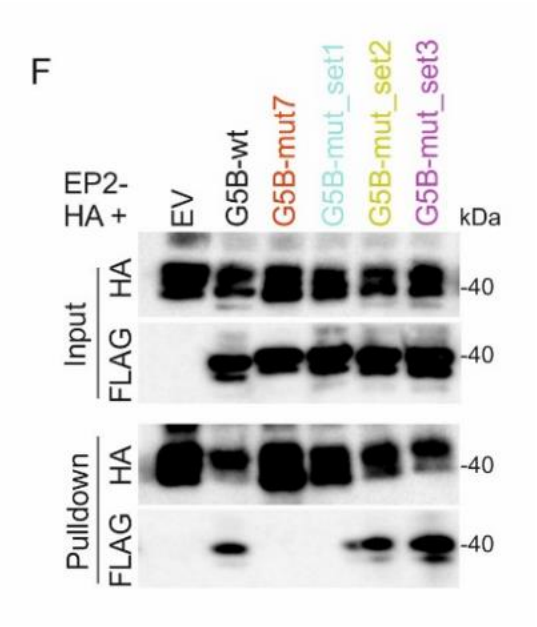
F
EP2-HA +
EV
G5B-wt
G5B-mut7
G5B-mut_set1
G5B-mut_set2
G5B-mut_set3
kDa
Input
HA
FLAG
Pulldown
HA
FLAG
-40
-40
-40
-40

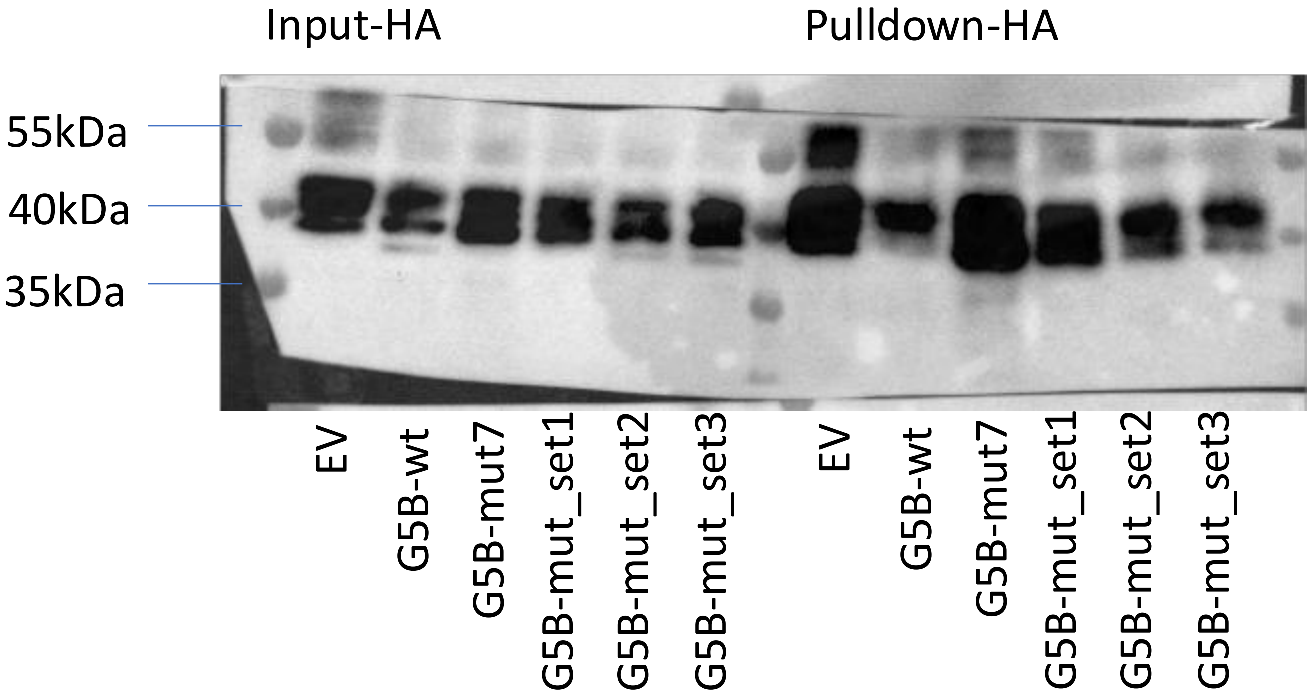
Input-HA
Pulldown-HA
55kDa
40kDa
35kDa
EV
G5B-wt
G5B-mut7
G5B-mut_set1
G5B-mut_set2
G5B-mut_set3
EV
G5B-wt
G5B-mut7
G5B-mut_set1
G5B-mut_set2
G5B-mut_set3

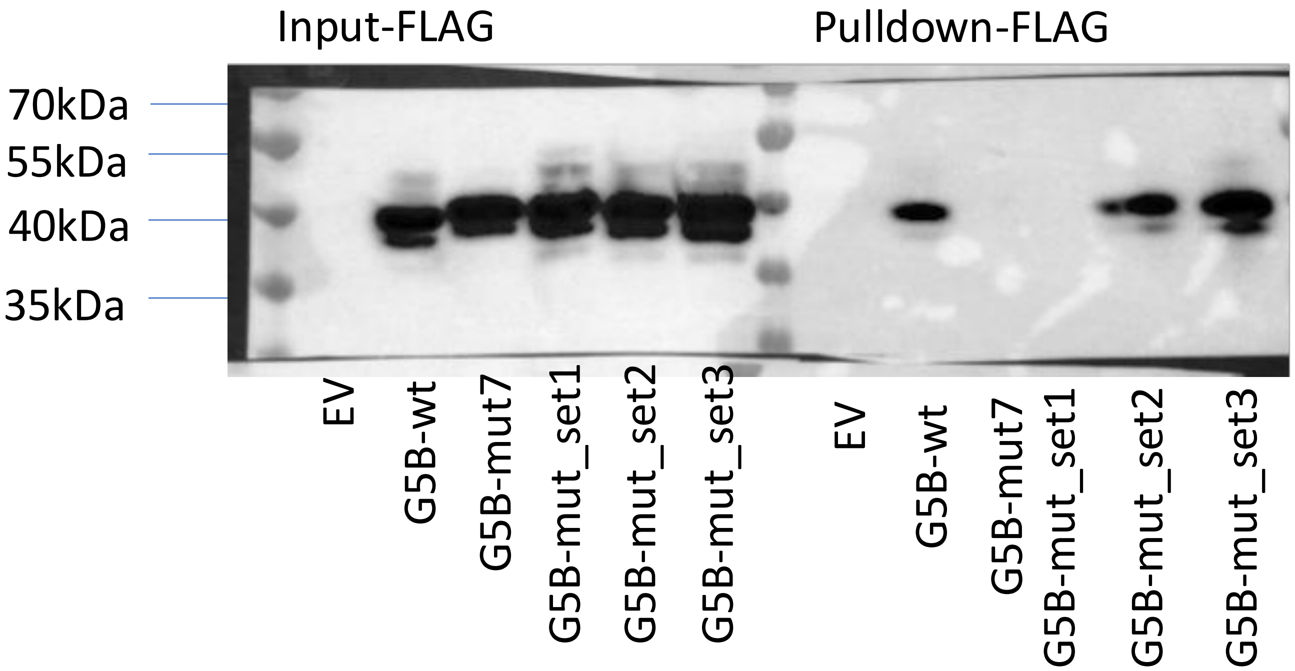
Input-FLAG
Pulldown-FLAG
70kDa
55kDa
40kDa
35kDa
EV
G5B-wt
G5B-mut7
G5B-mut_set1
G5B-mut_set2
G5B-mut_set3
EV
G5B-wt
G5B-mut7
G5B-mut_set1
G5B-mut_set2
G5B-mut_set3

Fig 7B

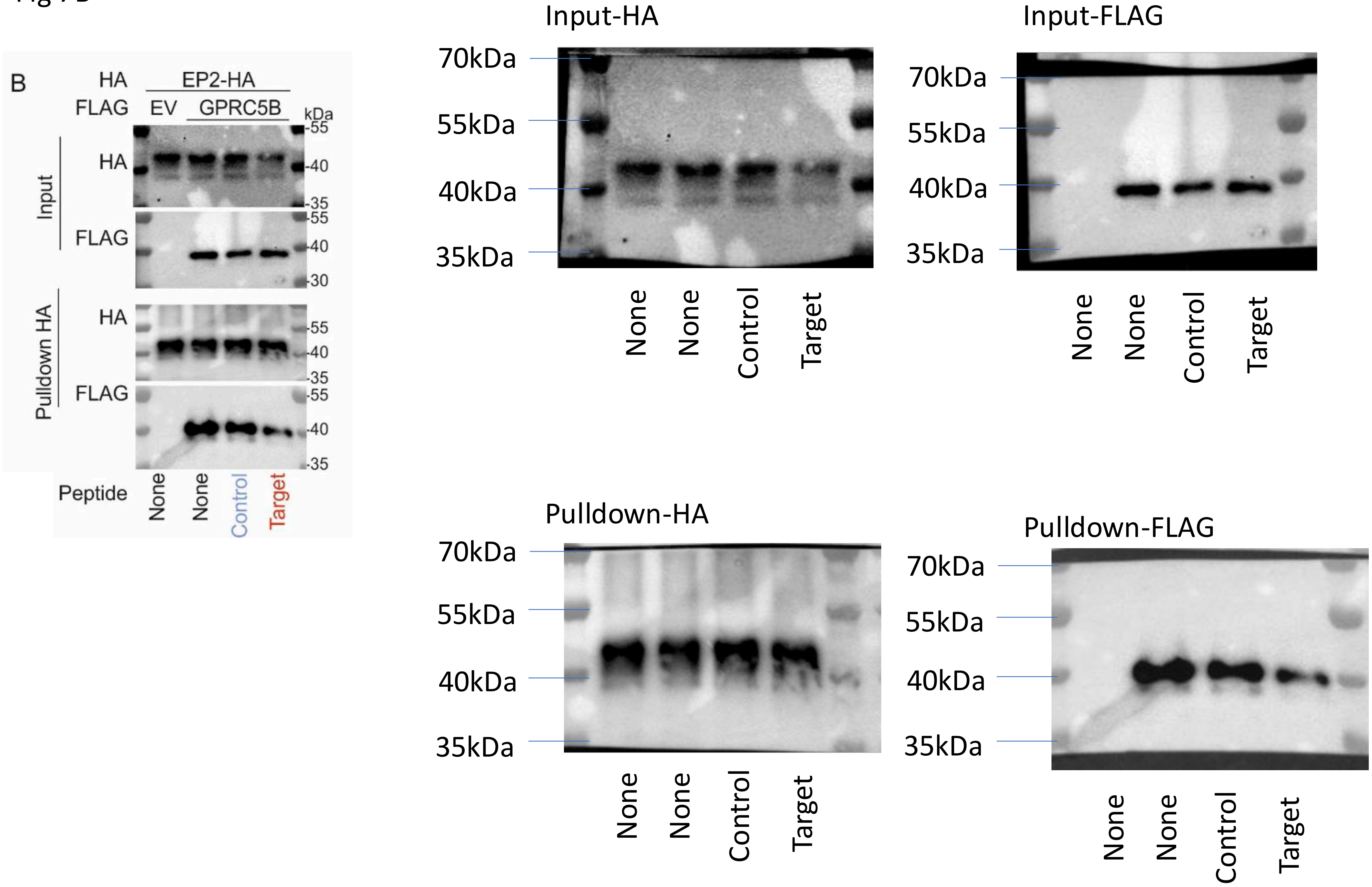
B
HA
EP2-HA
FLAG
EV
GPRC5B
kDa
Input
HA
FLAG
Pulldown HA
HA
FLAG
-55
-40
-35
-55
-40
-30
-55
-40
-35
-55
-40
-35
Peptide
None
None
Control
Target
Input-HA
70kDa
55kDa
40kDa
35kDa
None
None
Control
Target
Input-FLAG
70kDa
55kDa
40kDa
35kDa
None
None
Control
Target
Pulldown-HA
70kDa
55kDa
40kDa
35kDa
None
None
Control
Target
Pulldown-FLAG
70kDa
55kDa
40kDa
35kDa
None
None
Control
Target

Suppl. Fig 6A

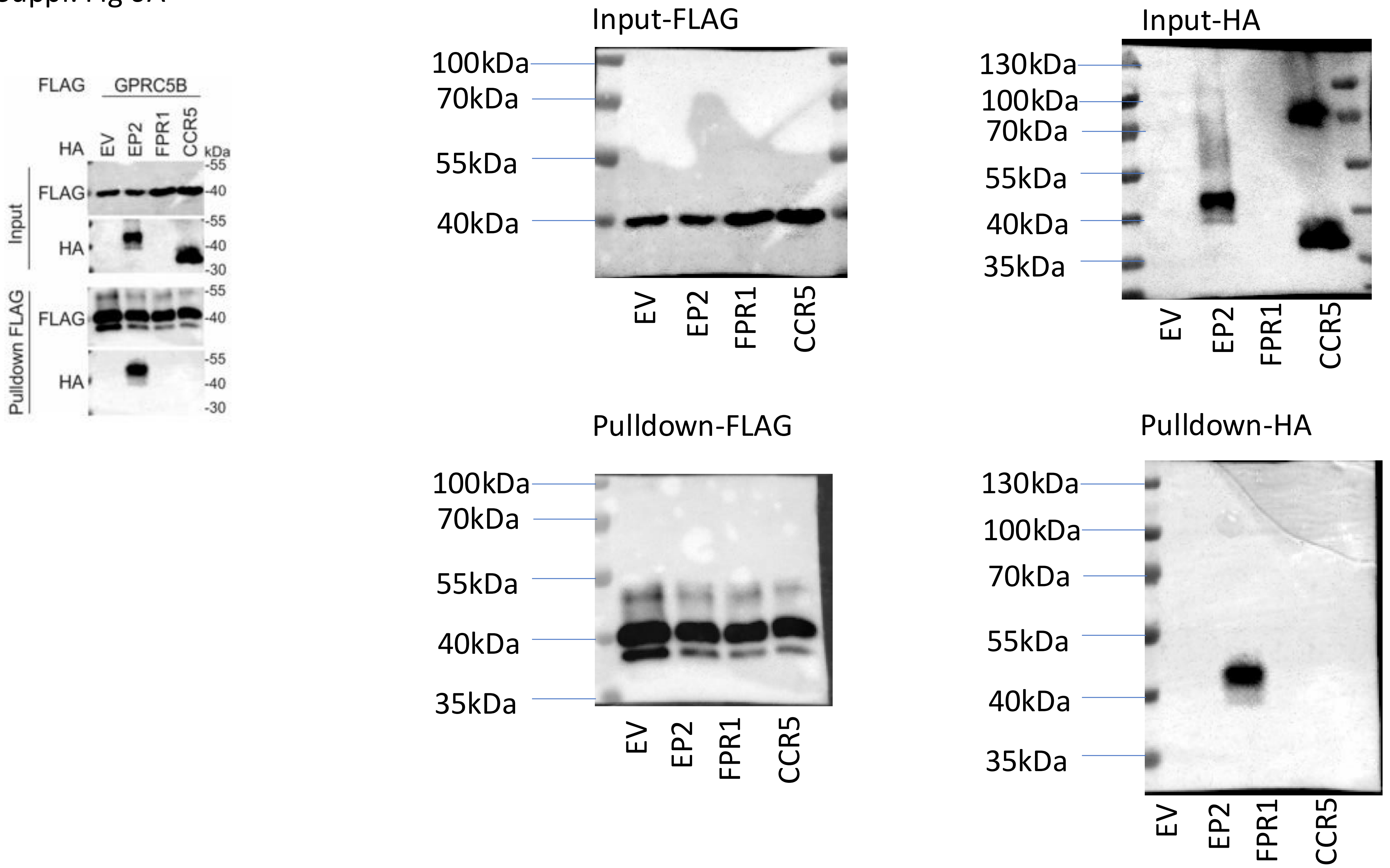

FLAG
GPRC5B
HA
EV
EP2
FPR1
CCR5
kDa
Input
FLAG
HA
Pulldown FLAG
-55
-40
-30
Input-FLAG
100kDa
70kDa
55kDa
40kDa
Input-HA
130kDa
100kDa
70kDa
55kDa
40kDa
35kDa
Pulldown-FLAG
100kDa
70kDa
55kDa
40kDa
35kDa
Pulldown-HA
130kDa
100kDa
70kDa
55kDa
40kDa
35kDa

Suppl. Fig 6B

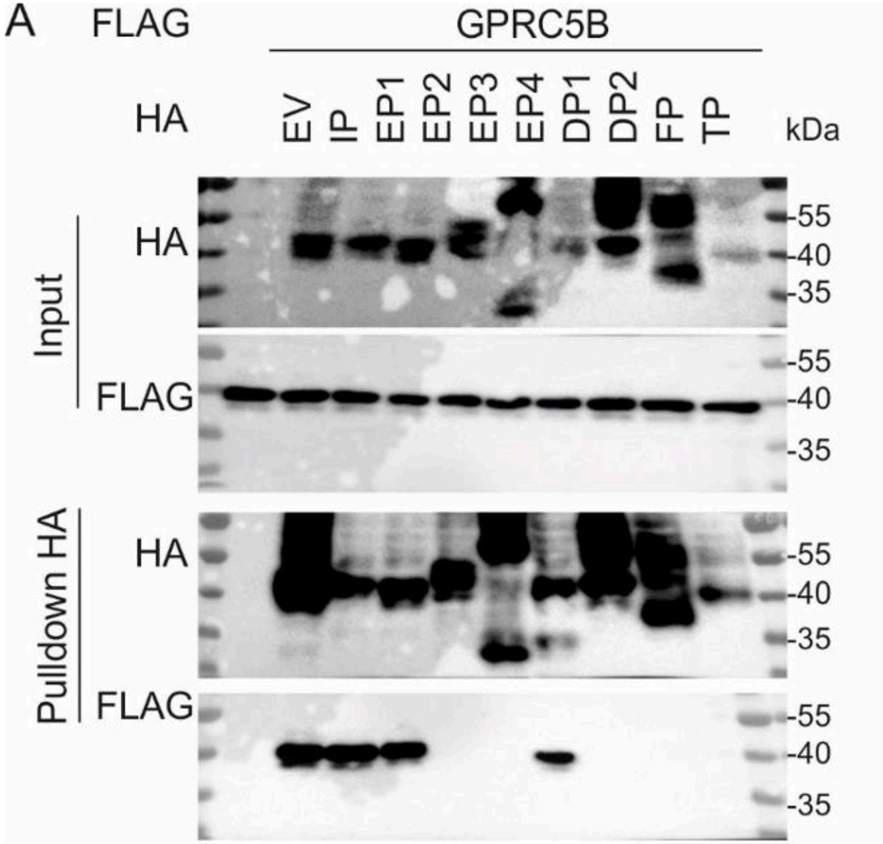
A
FLAG
GPRC5B
HA
EV
IP
EP1
EP2
EP3
EP4
DP1
DP2
FP
TP
kDa
Input
HA
FLAG
Pulldown HA
HA
FLAG
-55
-40
-35

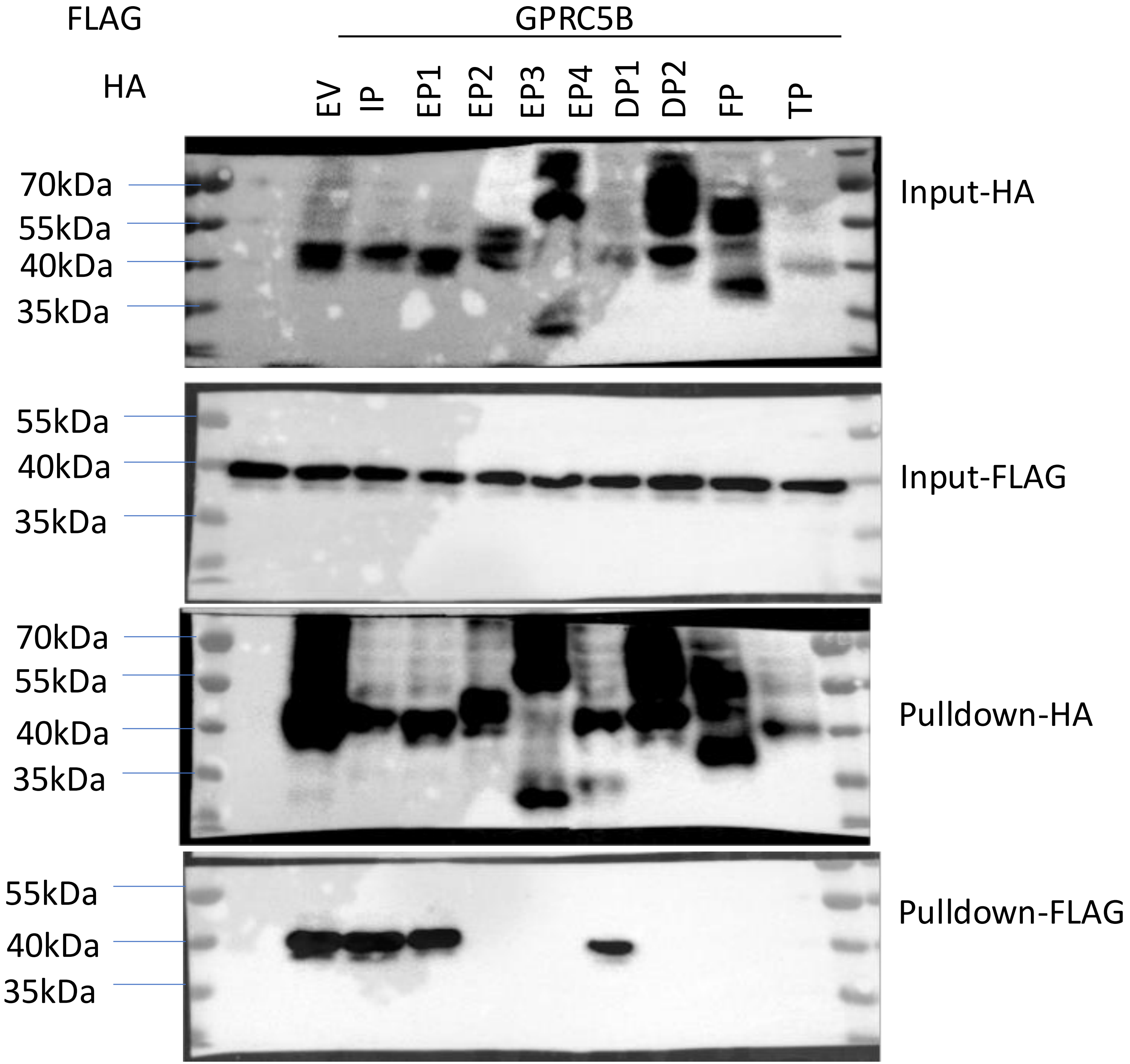
FLAG
GPRC5B
HA
EV
IP
EP1
EP2
EP3
EP4
DP1
DP2
FP
TP
70kDa
55kDa
40kDa
35kDa
Input-HA
Input-FLAG
Pulldown-HA
Pulldown-FLAG

Suppl. Fig 7E

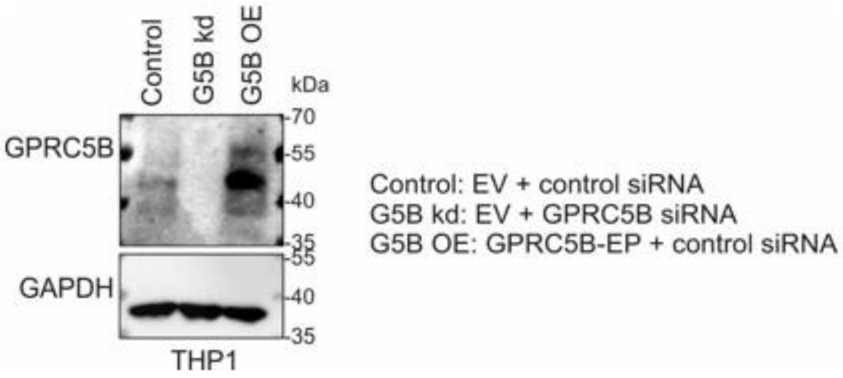
Control
G5B kd
G5B OE
kDa
GPRC5B
70
55
40
35
55
GAPDH
40
35
THP1
Control: EV + control siRNA
G5B kd: EV + GPRC5B siRNA
G5B OE: GPRC5B-EP + control siRNA

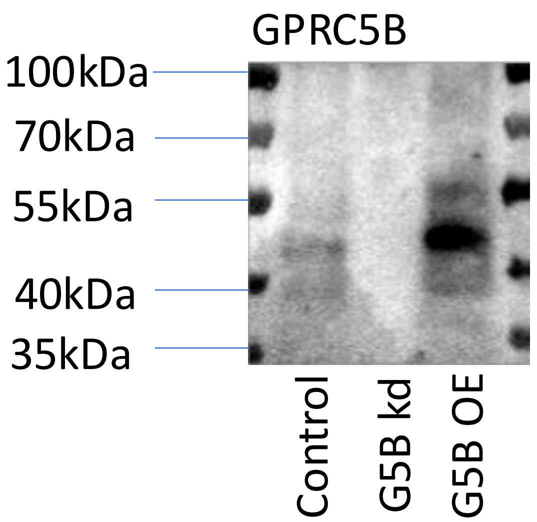
GPRC5B
100kDa
70kDa
55kDa
40kDa
35kDa
Control
G5B kd
G5B OE

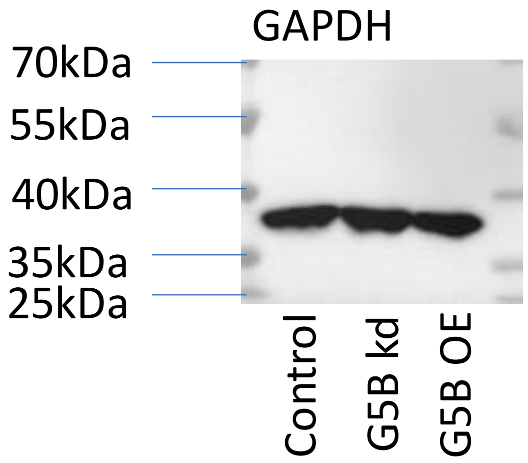
GAPDH
70kDa
55kDa
40kDa
35kDa
25kDa
Control
G5B kd
G5B OE

Suppl. Fig 8A

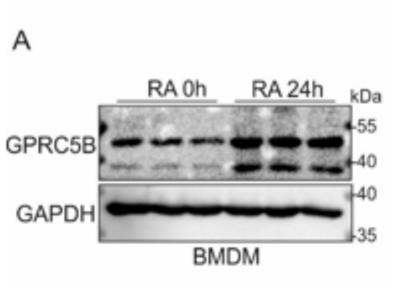
A
RA 0h
RA 24h
kDa
GPRC5B
55
40
40
GAPDH
35
BMDM

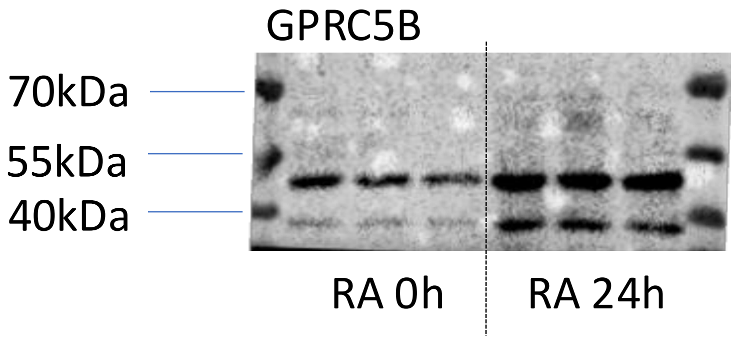
GPRC5B
70kDa
55kDa
40kDa
RA 0h
RA 24h

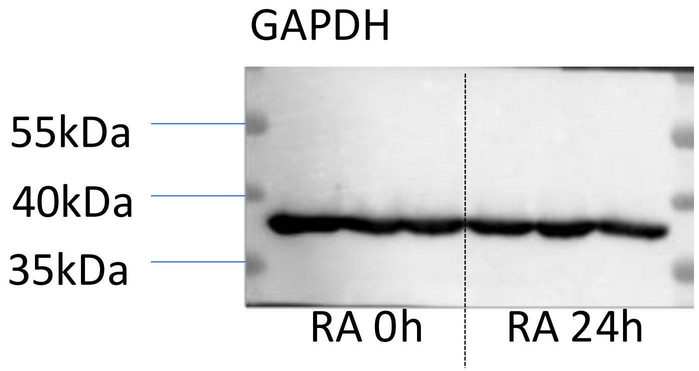
GAPDH
55kDa
40kDa
35kDa
RA 0h
RA 24h

Suppl. Fig 8C

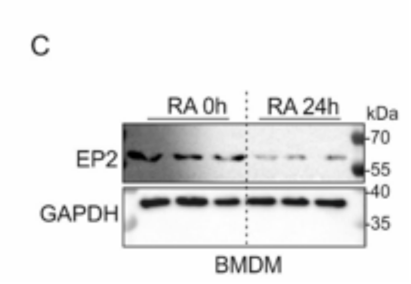
C
RA 0h
RA 24h
kDa
70
55
40
35
EP2
GAPDH
BMDM

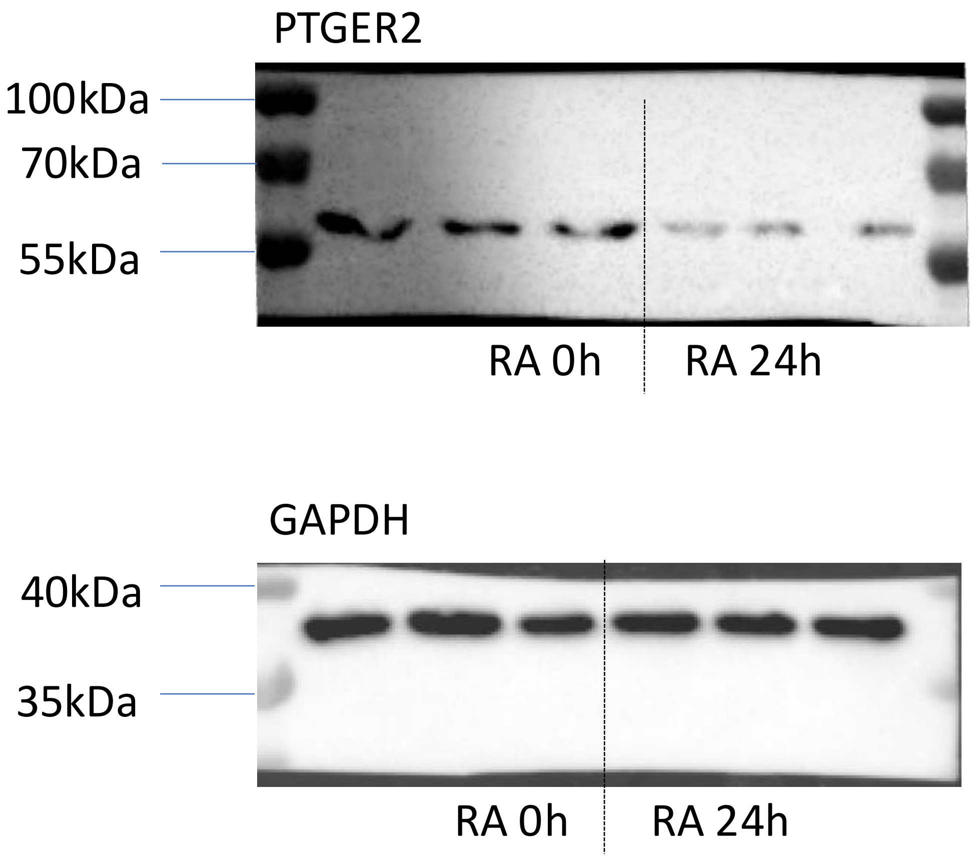
PTGER2
100kDa
70kDa
55kDa
RA 0h
RA 24h
GAPDH
40kDa
35kDa
RA 0h
RA 24h

Suppl. Fig 8F

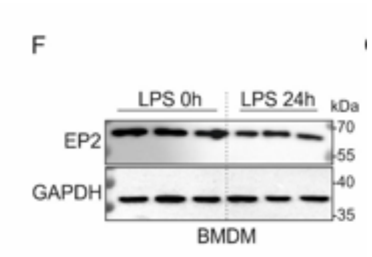
F
LPS 0h
LPS 24h
kDa
EP2
GAPDH
70
55
40
35
BMDM

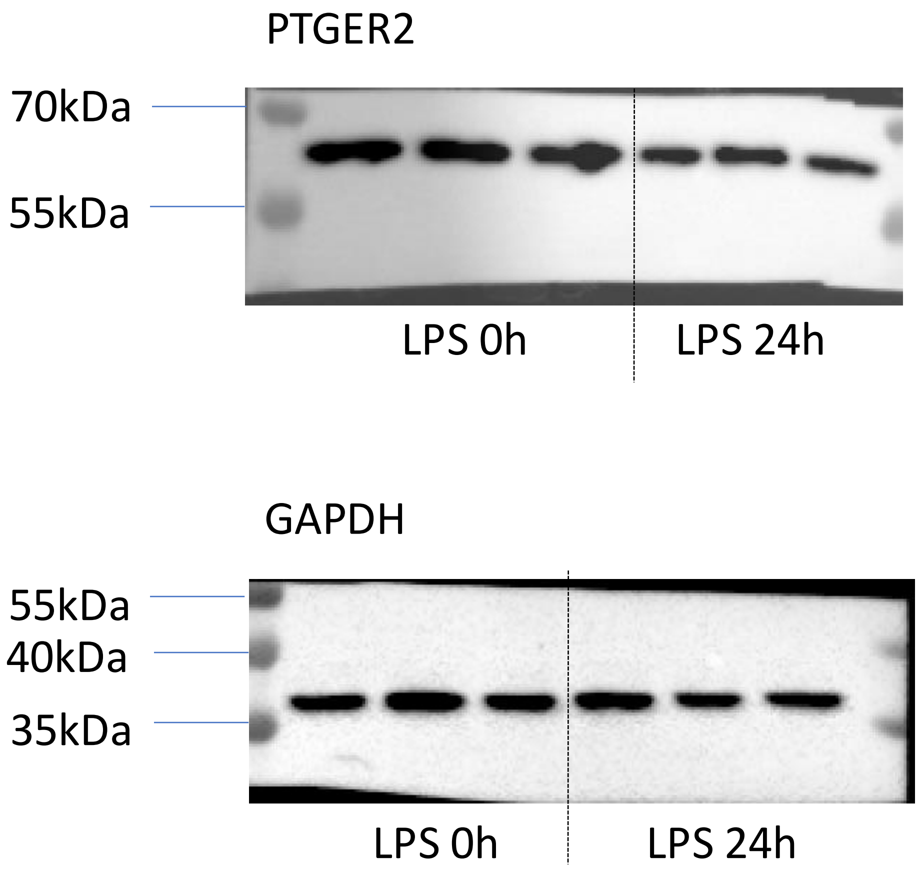
PTGER2
70kDa
55kDa
LPS 0h
LPS 24h
GAPDH
55kDa
40kDa
35kDa
LPS 0h
LPS 24h

Suppl. Fig 10A

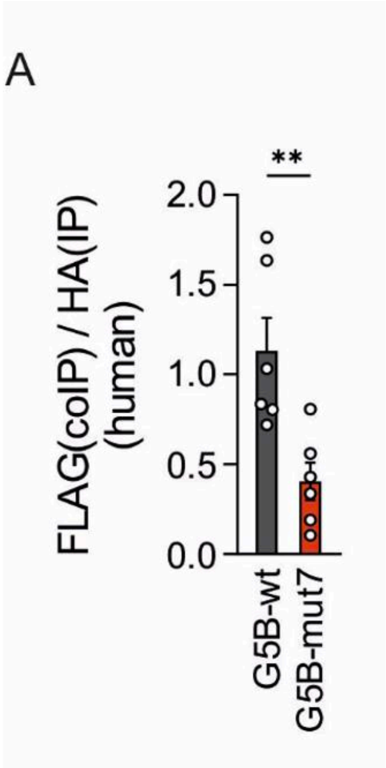

Blots used for quantification in Suppl. Fig 10A

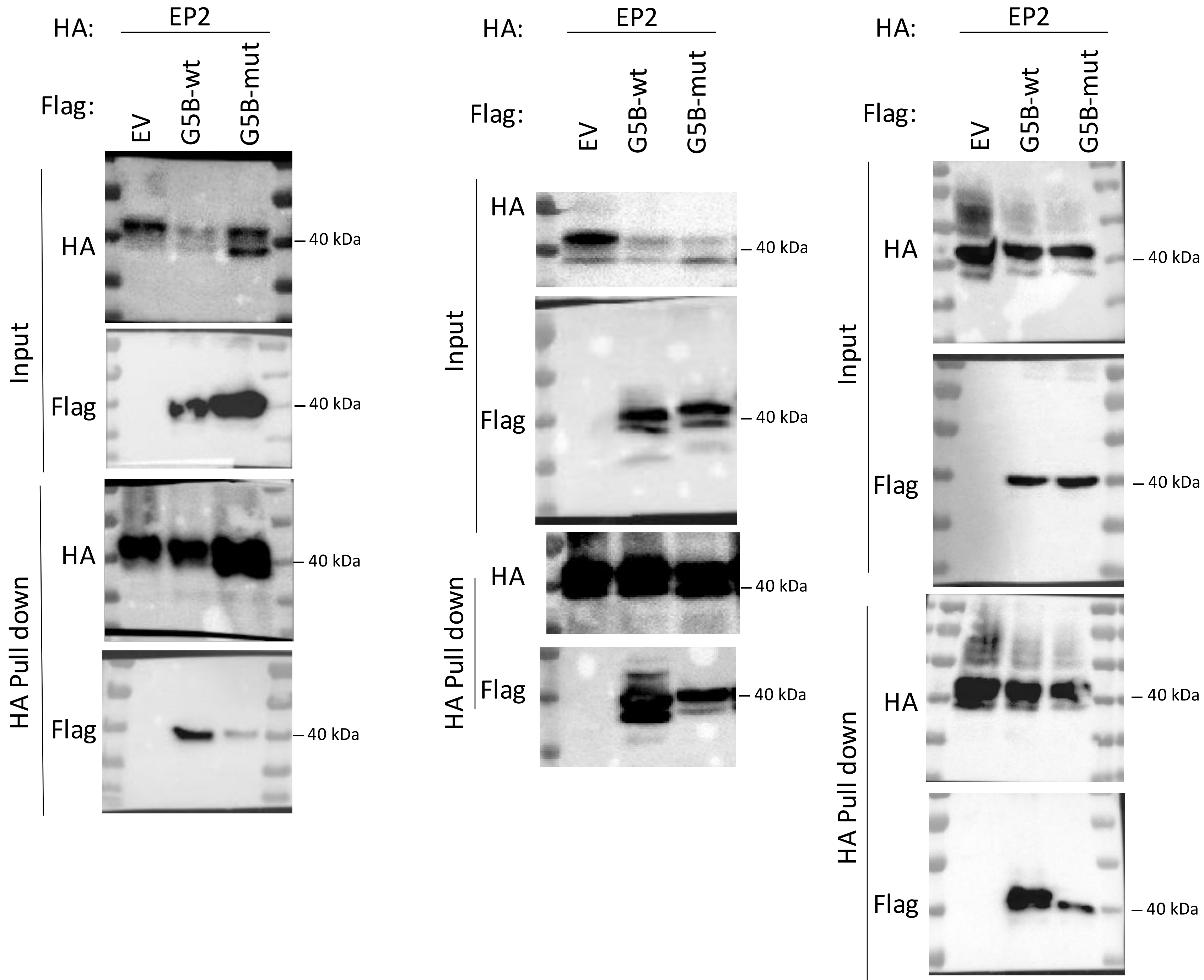

Suppl. Fig 10A

Blots used for quantification in Suppl. Fig 10A

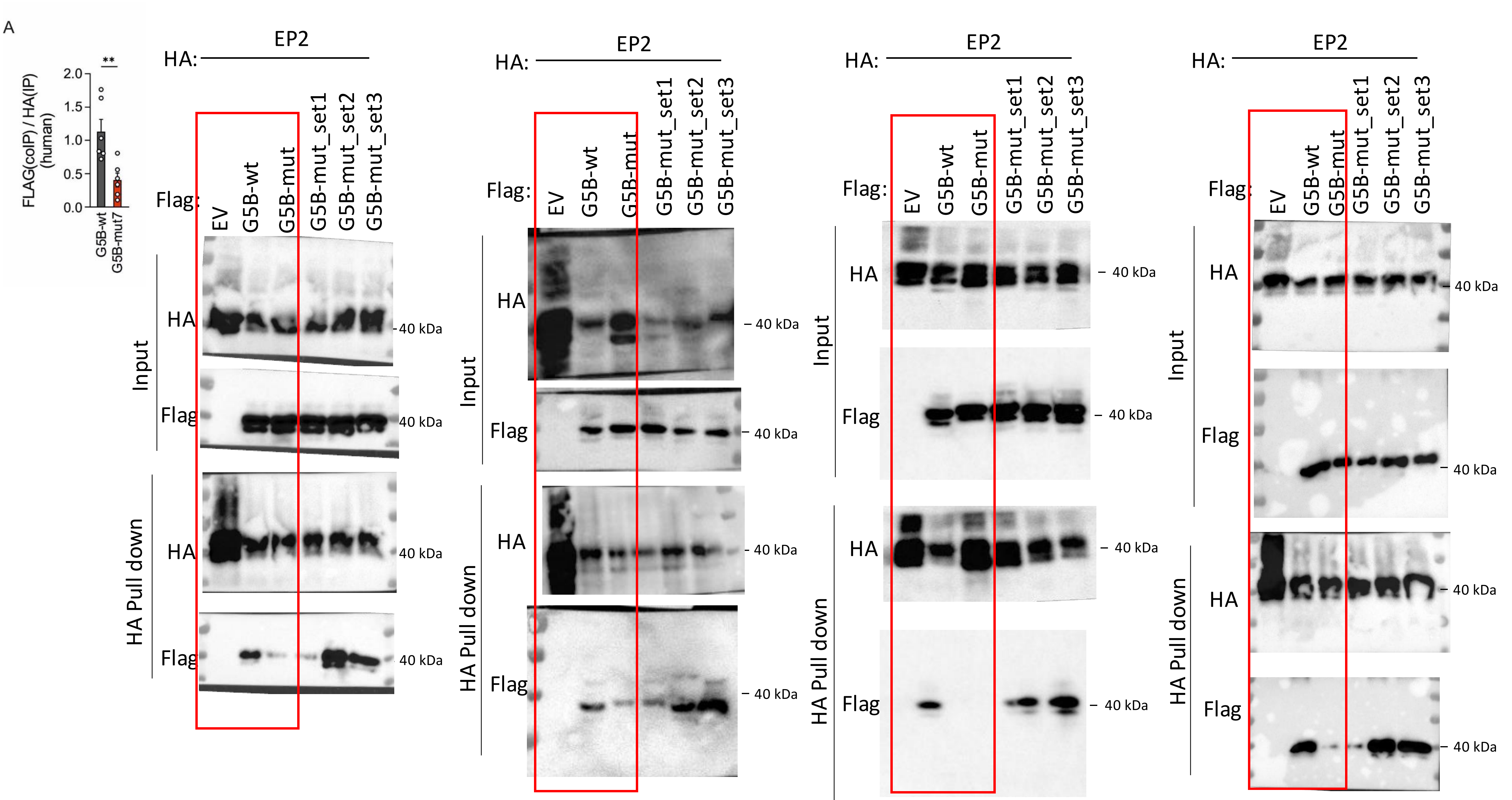

Suppl. Fig 10B

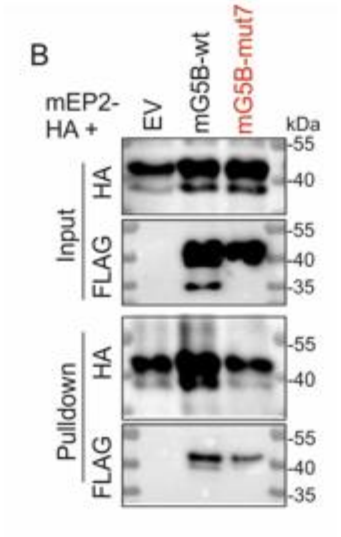

Input-HA

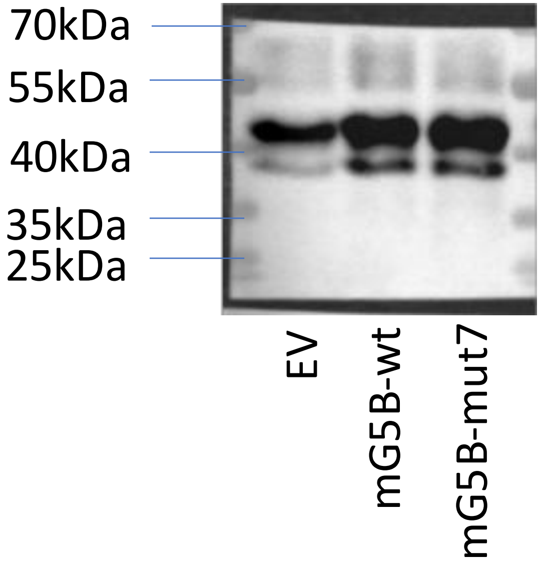

Pulldown-HA

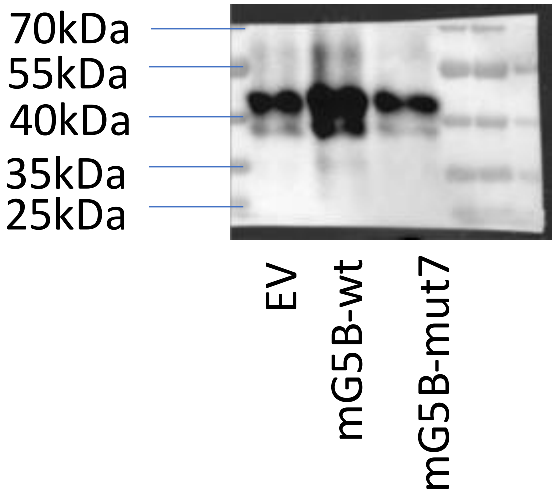

Input-FLAG

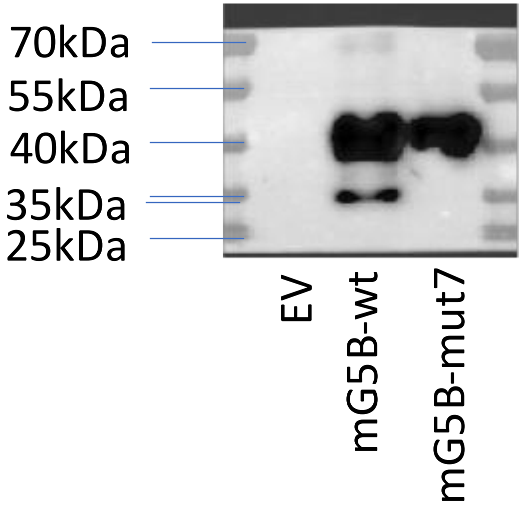

Pulldown-FLAG

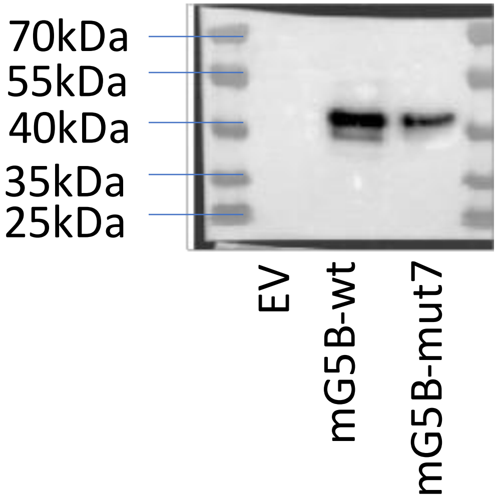

Suppl. Fig. 10C Blots used for quantification in Suppl. Fig 10C

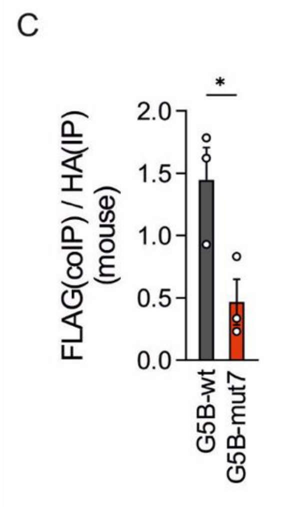

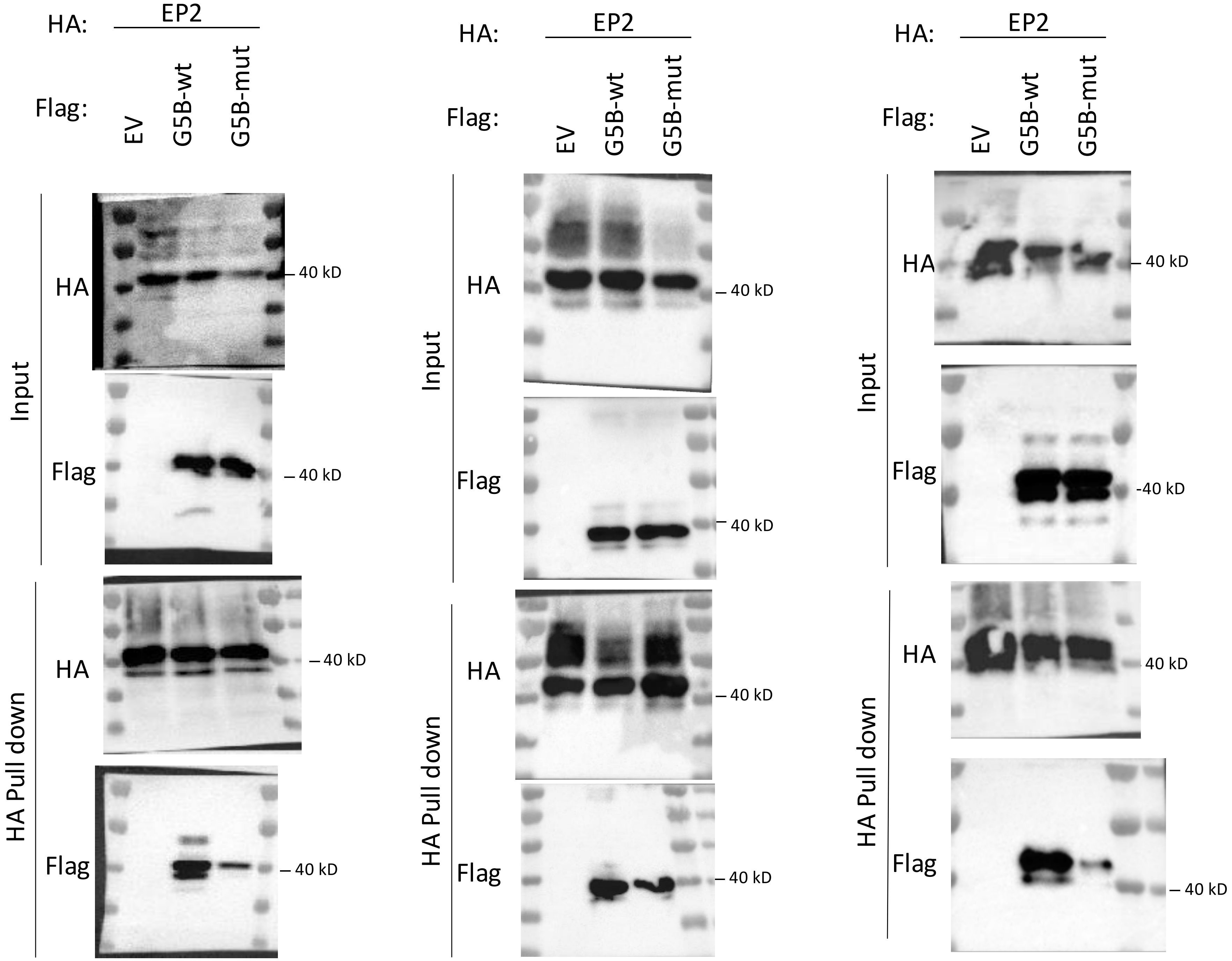

Suppl. Fig 10E

Blots used for quantification in Suppl. Fig 10E

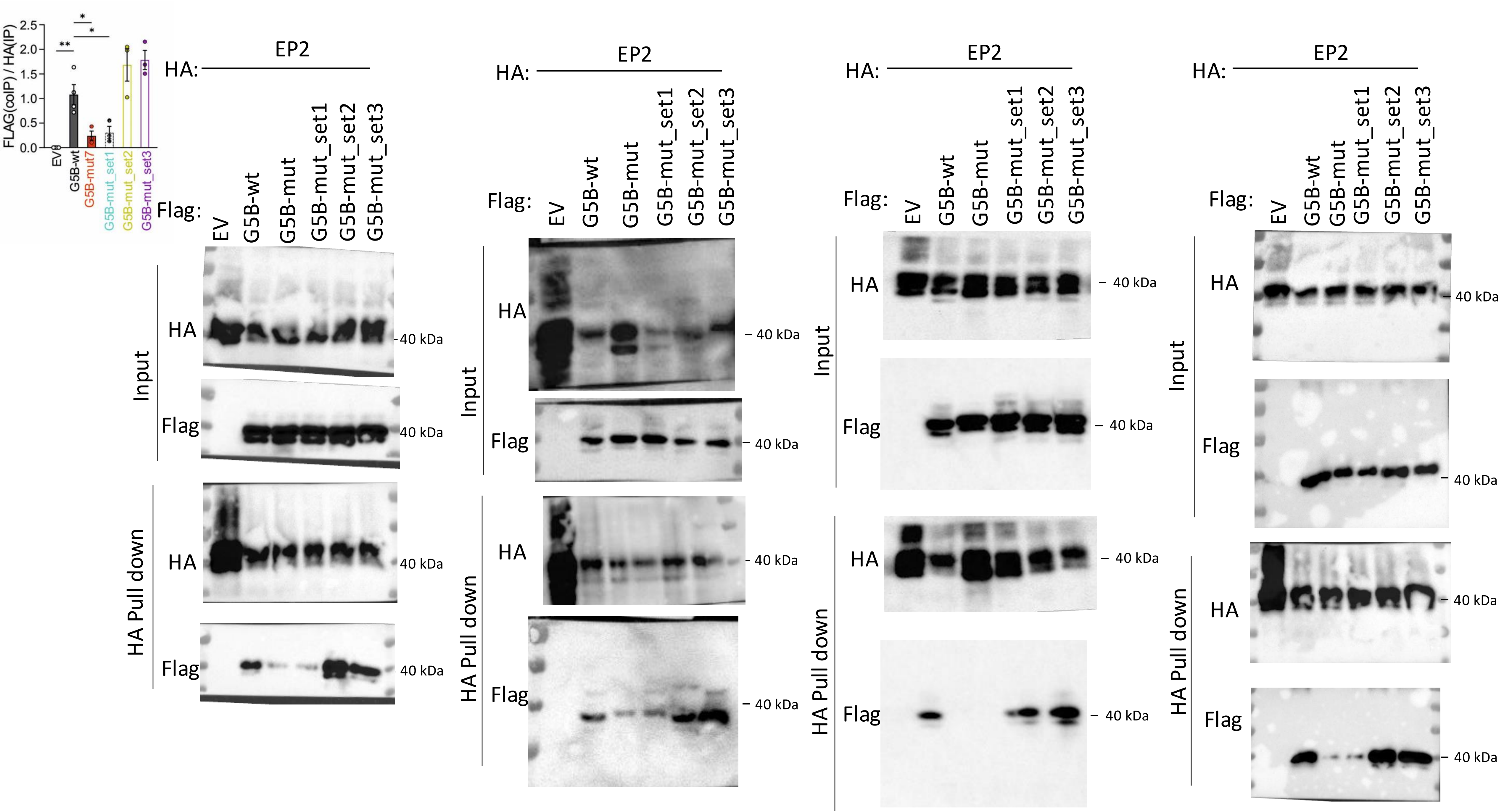

Suppl. Fig 11A

Blots used for quantification in Suppl. Fig 11A

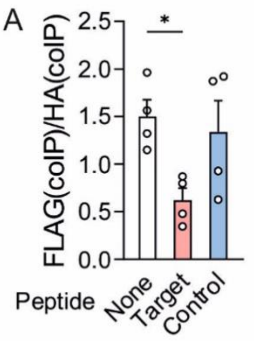

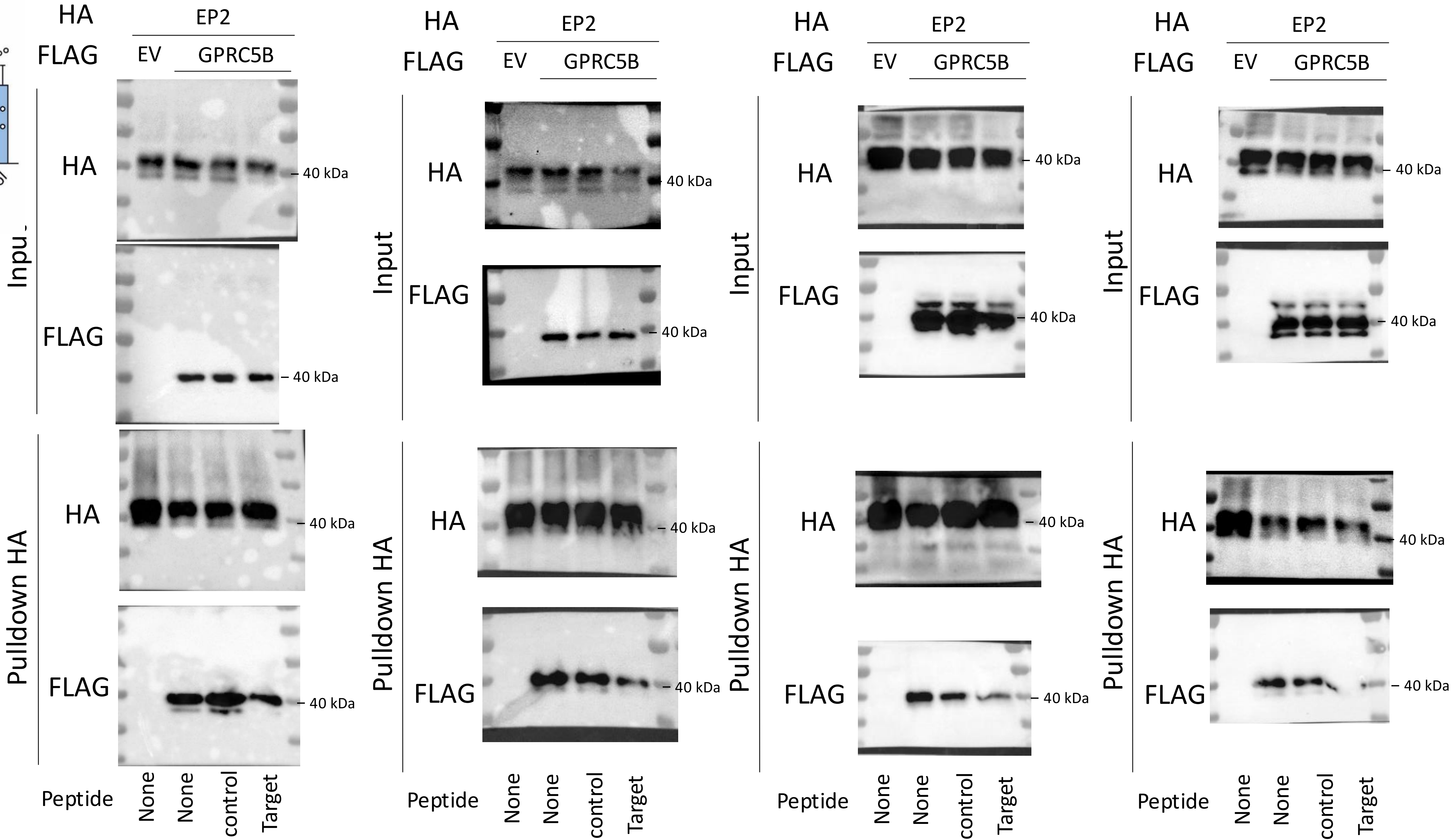

4. FACS gating strategies for Kwon et al., 2024

Figure 1O and P

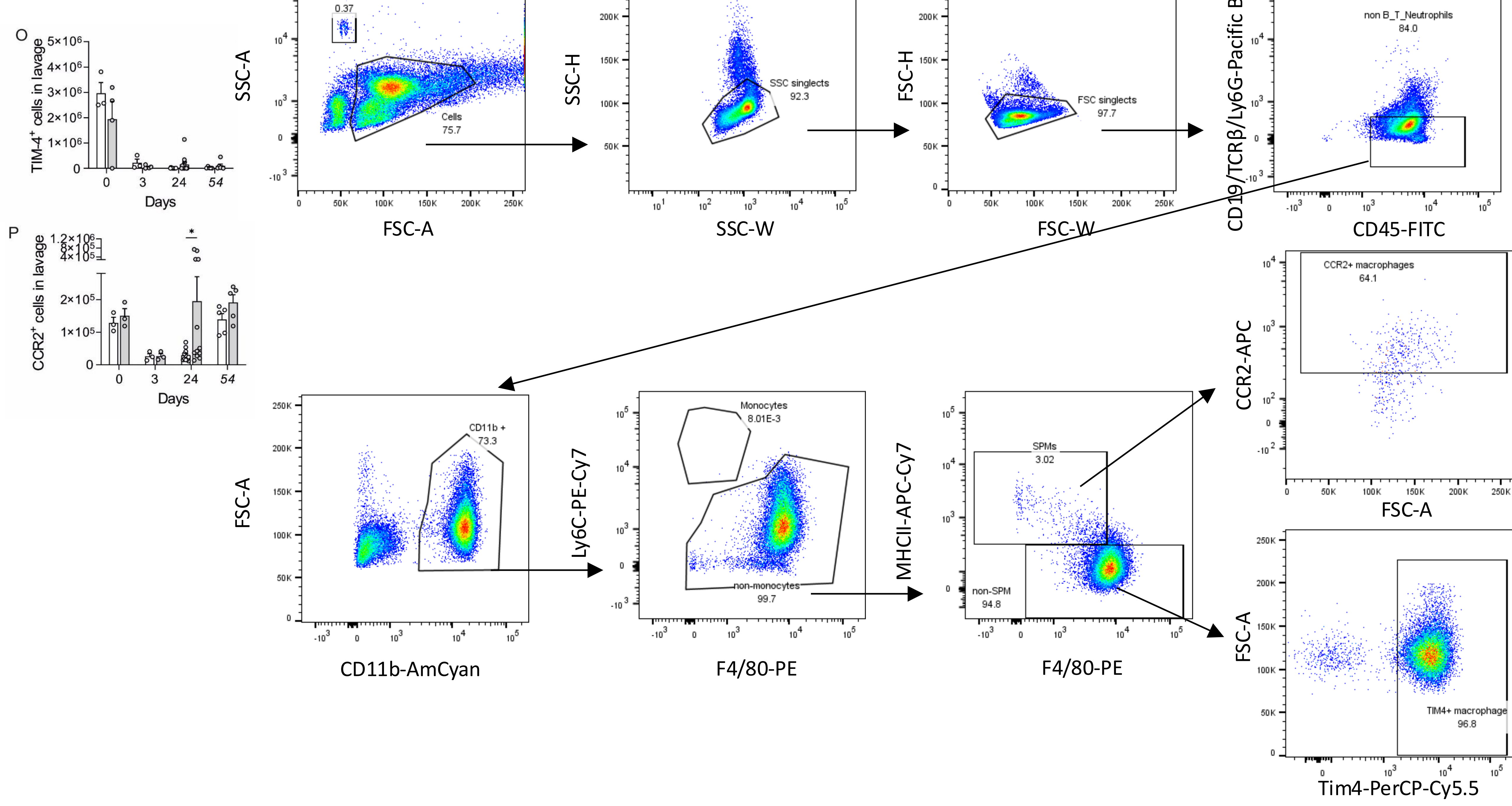

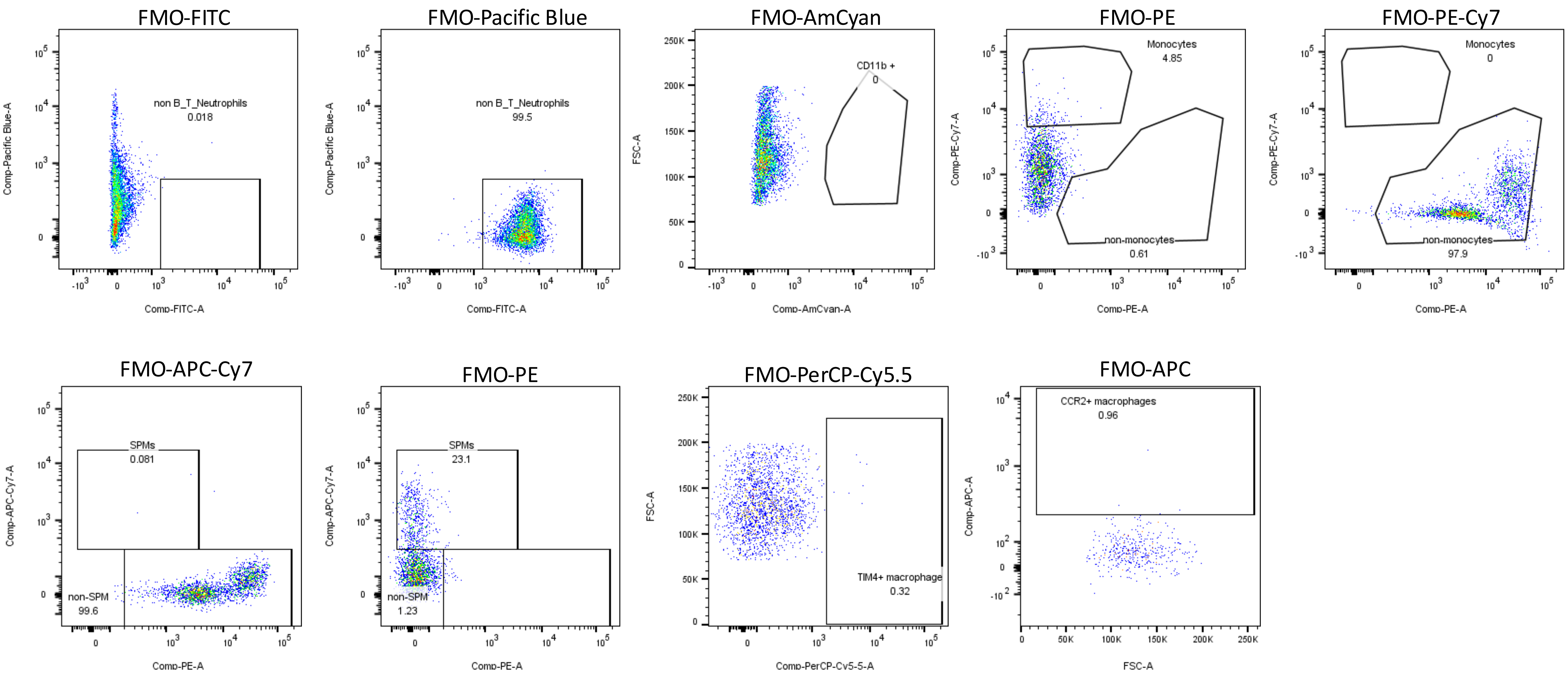
FMO-FITC
non B_T_Neutrophils
0.018
Comp-Pacific Blue-A
Comp-FITC-A
FMO-Pacific Blue
non B_T_Neutrophils
99.5
Comp-Pacific Blue-A
Comp-FITC-A
FMO-AmCyan
CD11b +
0
FSC-A
Comp-AmCyan-A
FMO-PE
Monocytes
4.85
non-monocytes
0.61
Comp-PE-Cy7-A
Comp-PE-A
FMO-PE-Cy7
Monocytes
0
non-monocytes
97.9
Comp-PE-Cy7-A
Comp-PE-A
FMO-APC-Cy7
SPMs
0.081
non-SPM
99.6
Comp-APC-Cy7-A
Comp-PE-A
FMO-PE
SPMs
23.1
non-SPM
1.23
Comp-APC-Cy7-A
Comp-PE-A
FMO-PerCP-Cy5.5
TIM4+ macrophage
0.32
FSC-A
Comp-PerCP-Cy5-5-A
FMO-APC
CCR2+ macrophages
0.96
Comp-APC-A
FSC-A

Figure 2I

Suppl. Figure 2E and F

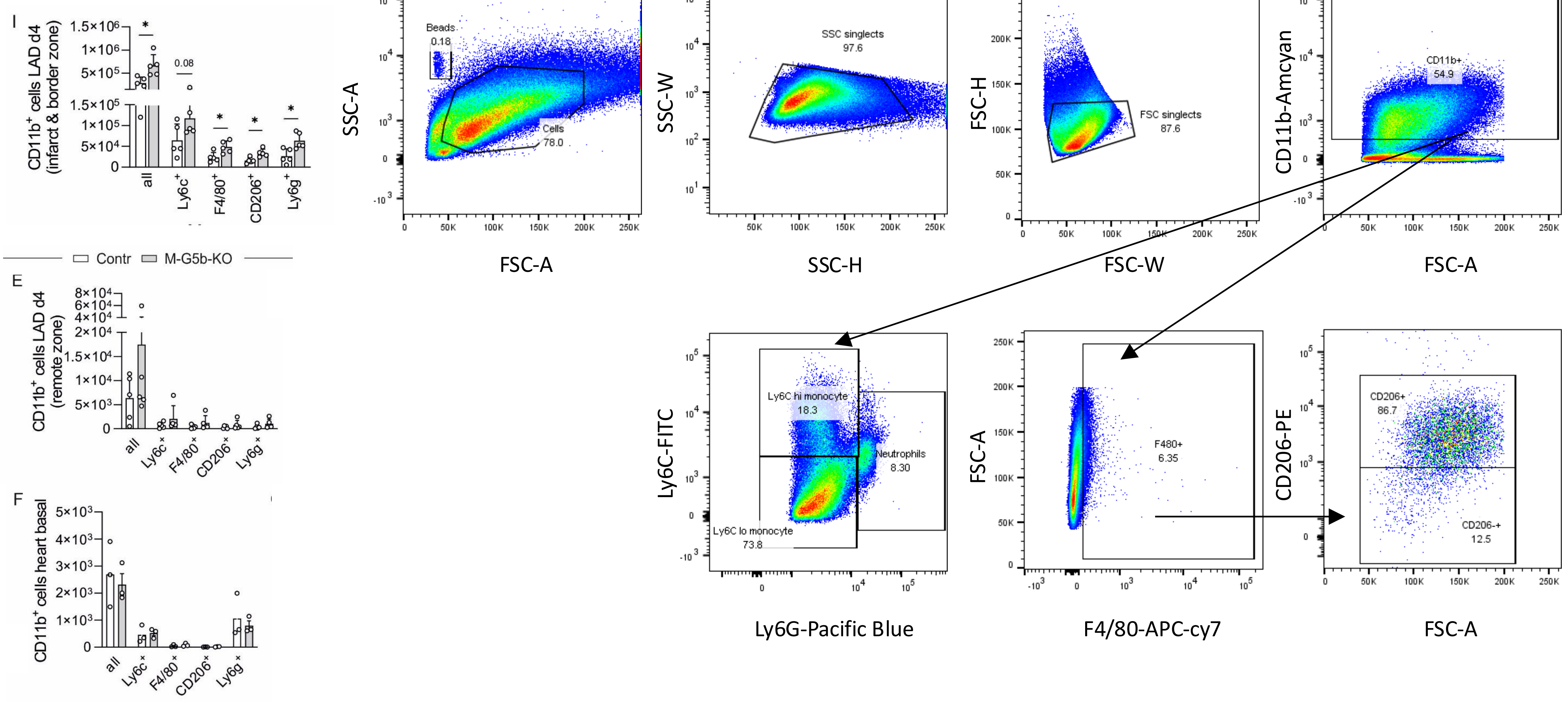

FMO-FITC FMO-PE FMO-APC-cy7 FMO-Pacificblue FMO-Amcyan

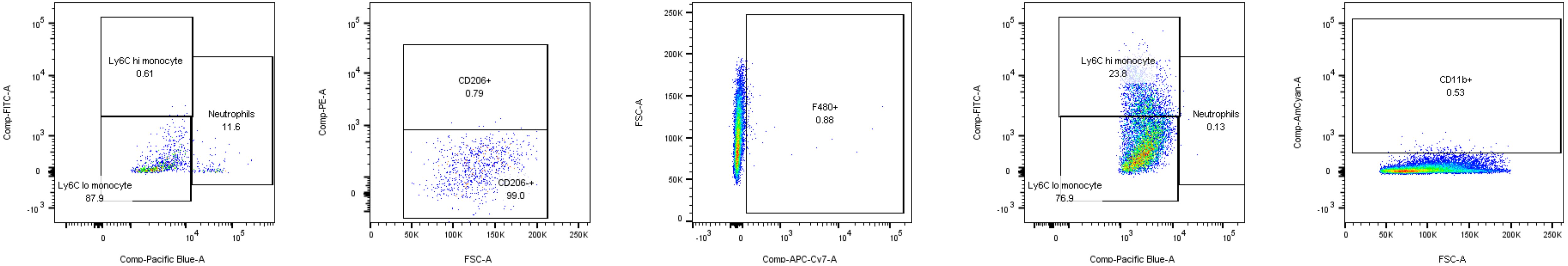

Suppl. Figure 1A-F

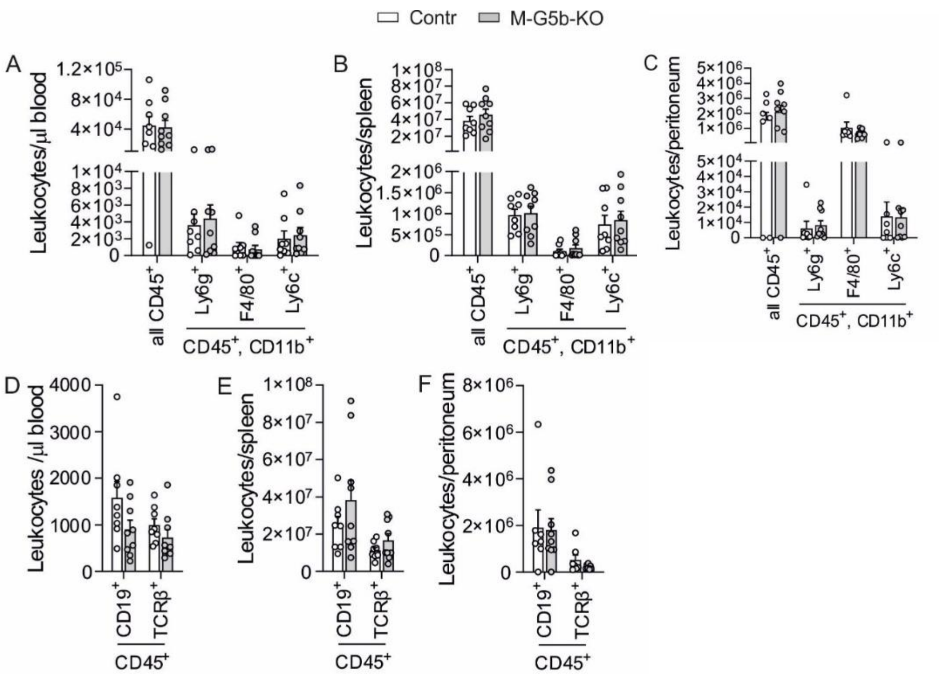

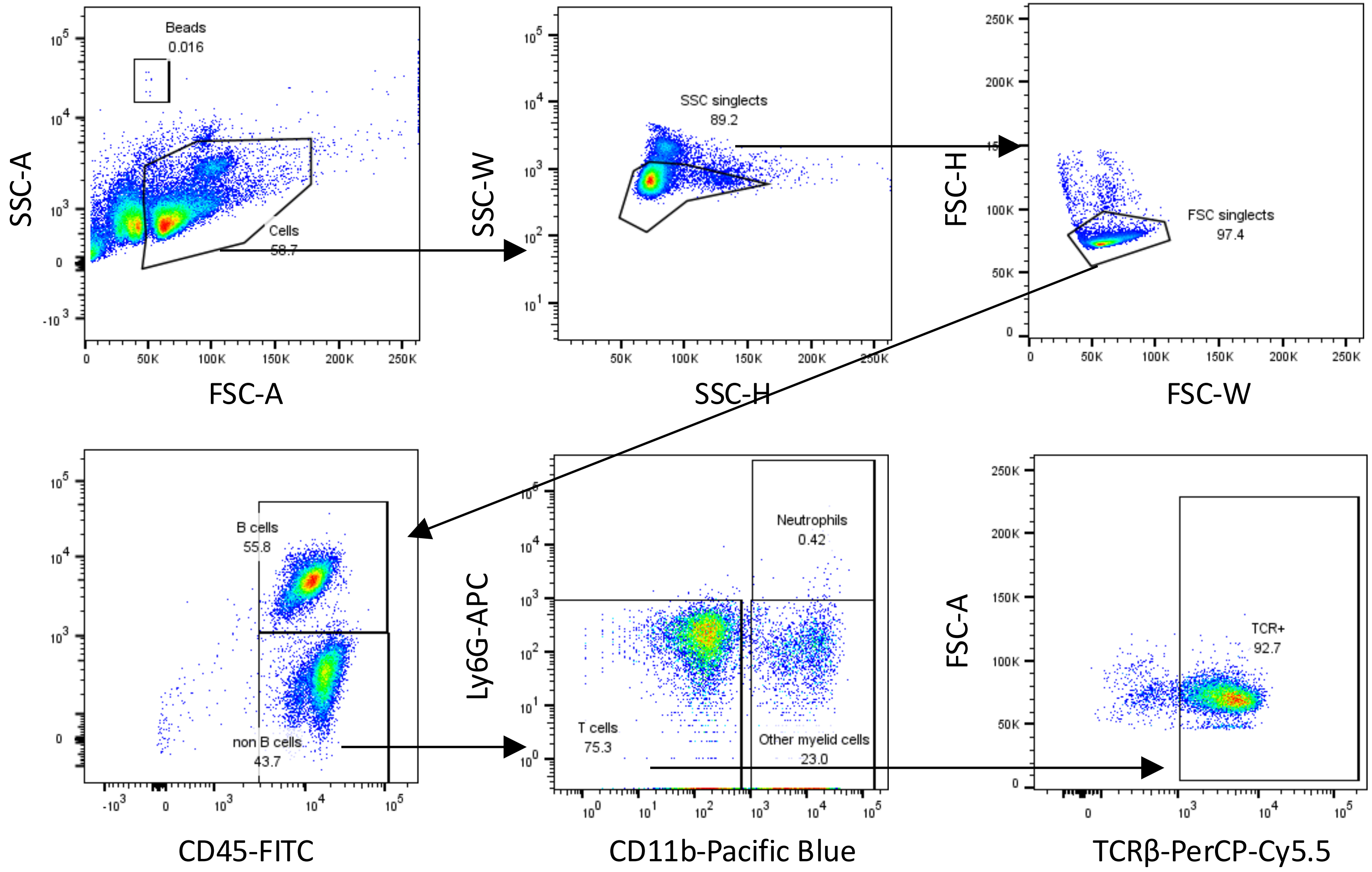

FMO-FITC

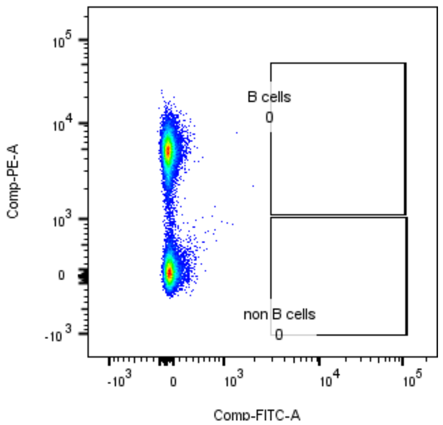

FMO-PE

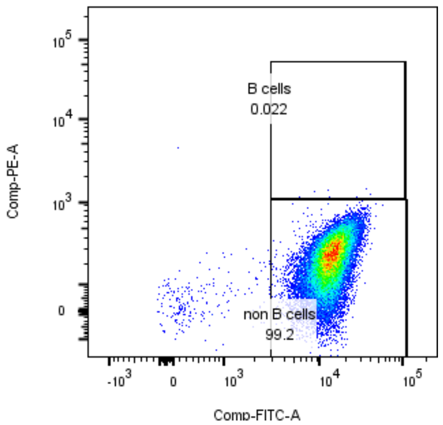

FMO-PerCP-Cy5.5

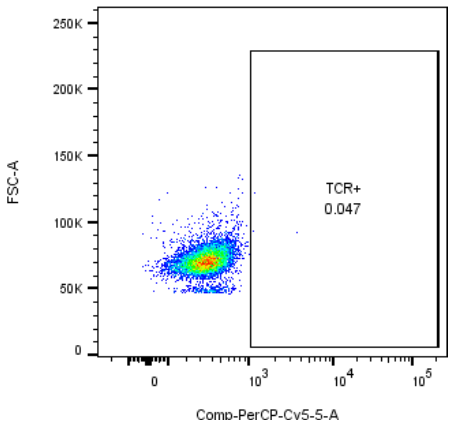

FMO-Pacific Bule

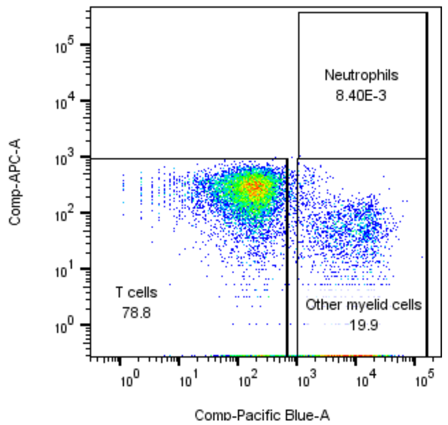

FMO-APC

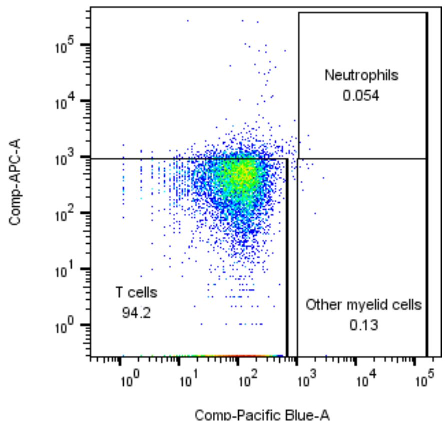

Suppl. Figure 1A-F

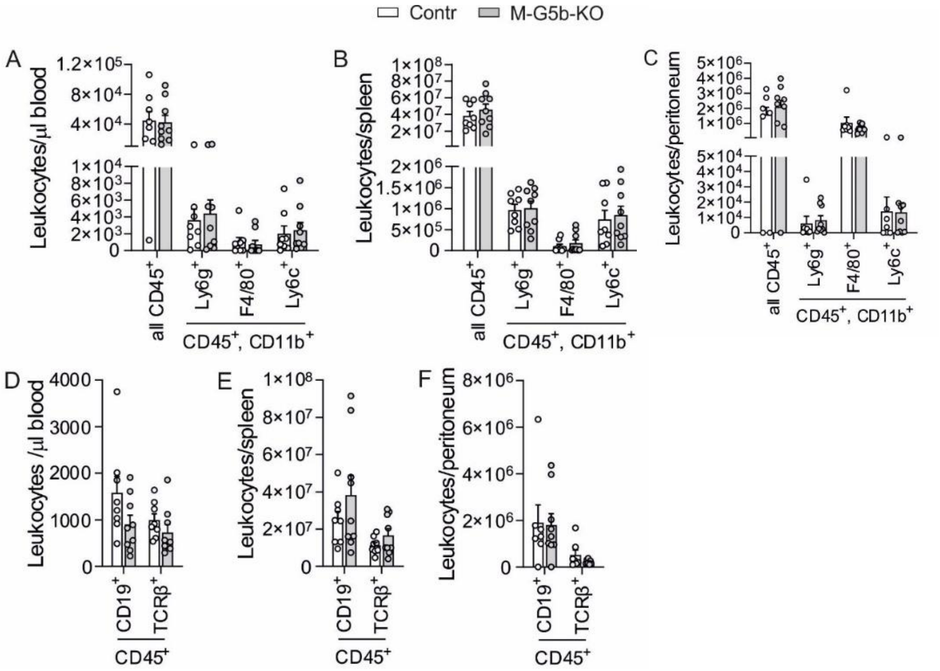

Myeloid cells

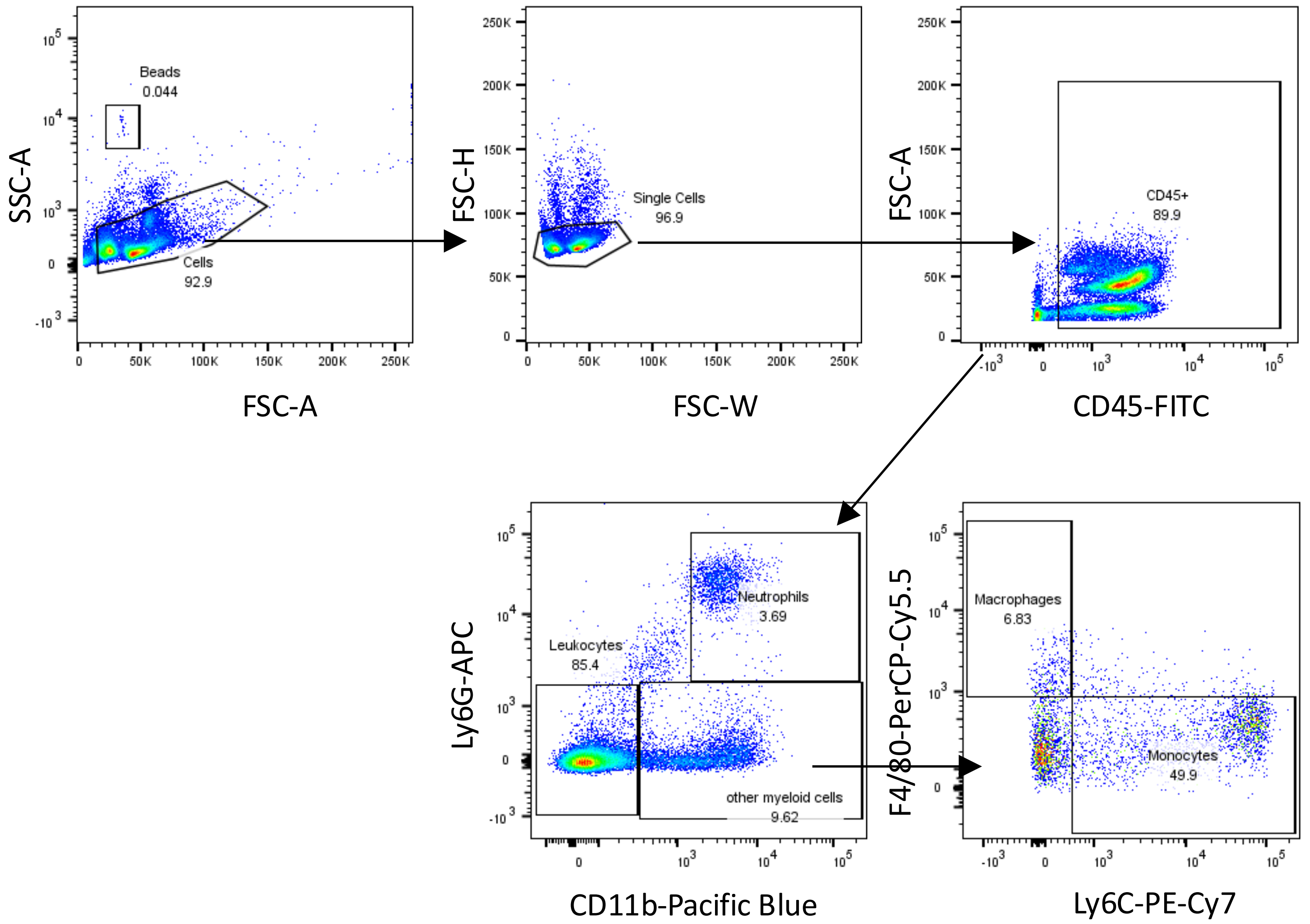

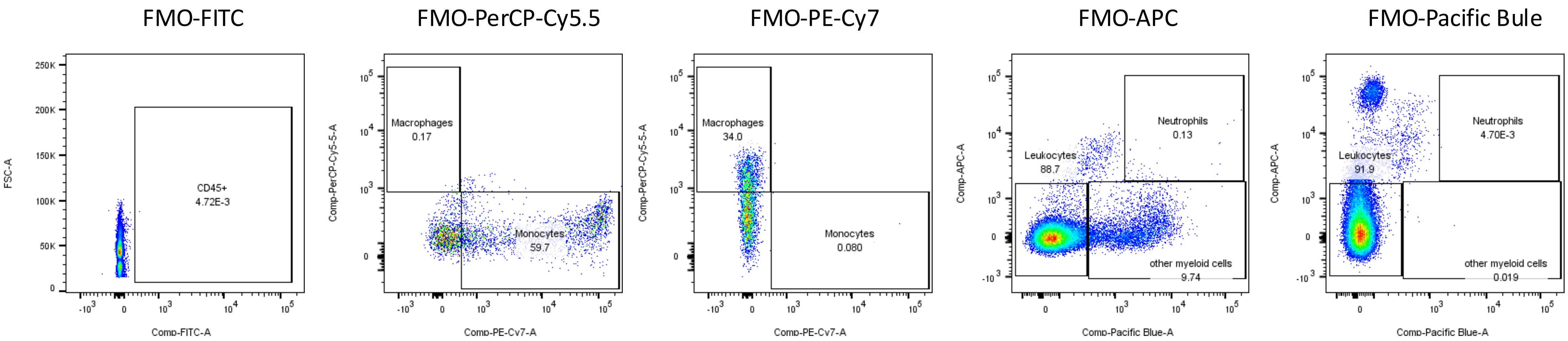
FMO-FITC
FMO-PerCP-Cy5.5
FMO-PE-Cy7
FMO-APC
FMO-Pacific Bule
CD45+
4.72E-3
FSC-A
Comp-FITC-A
Macrophages
0.17
Monocytes
59.7
Comp-PerCP-Cy5-5-A
Comp-PE-Cy7-A
Macrophages
34.0
Monocytes
0.080
Comp-PerCP-Cy5-5-A
Comp-PE-Cy7-A
Neutrophils
0.13
Leukocytes
88.7
other myeloid cells
9.74
Comp-APC-A
Comp-Pacific Blue-A
Neutrophils
4.70E-3
Leukocytes
91.9
other myeloid cells
0.019
Comp-APC-A
Comp-Pacific Blue-A

Suppl. Figure 1K and L

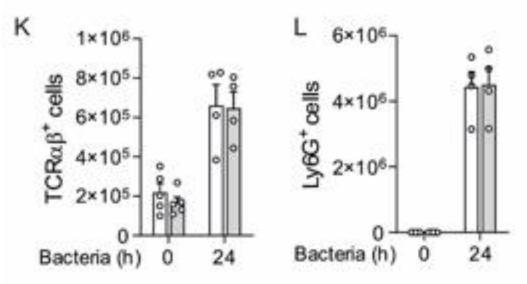

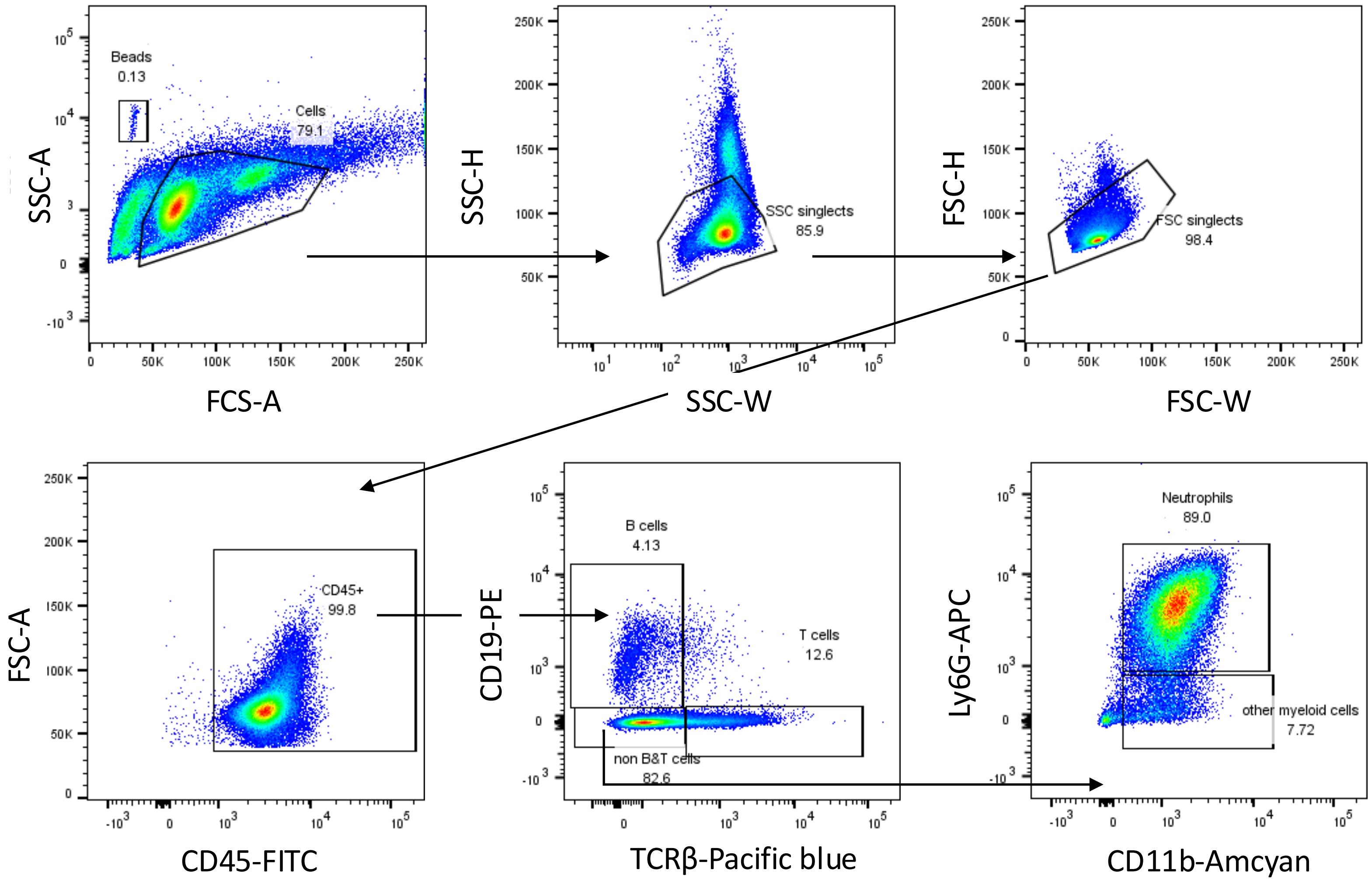

FMO-FITC FMO-PE FMO-Pacific blue FMO-Amcyan FMO-APC

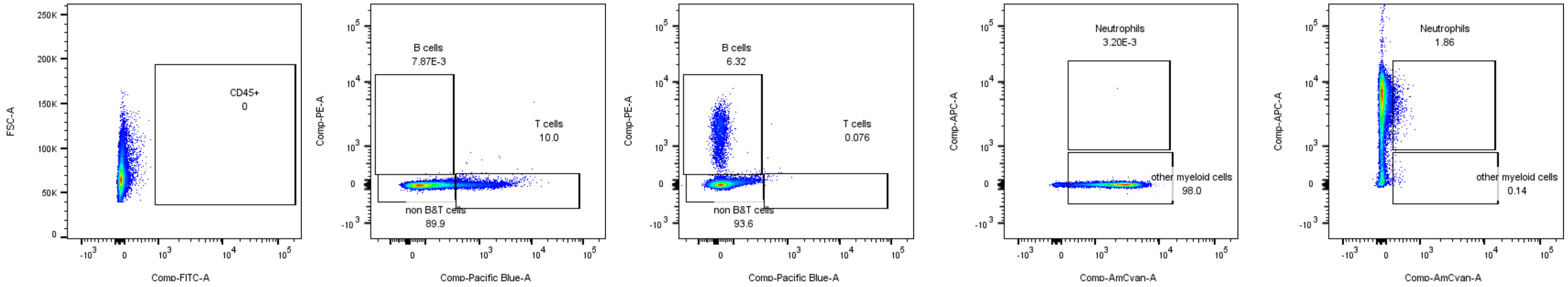

Suppl. Figure 2G

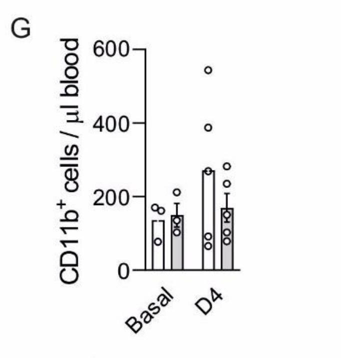

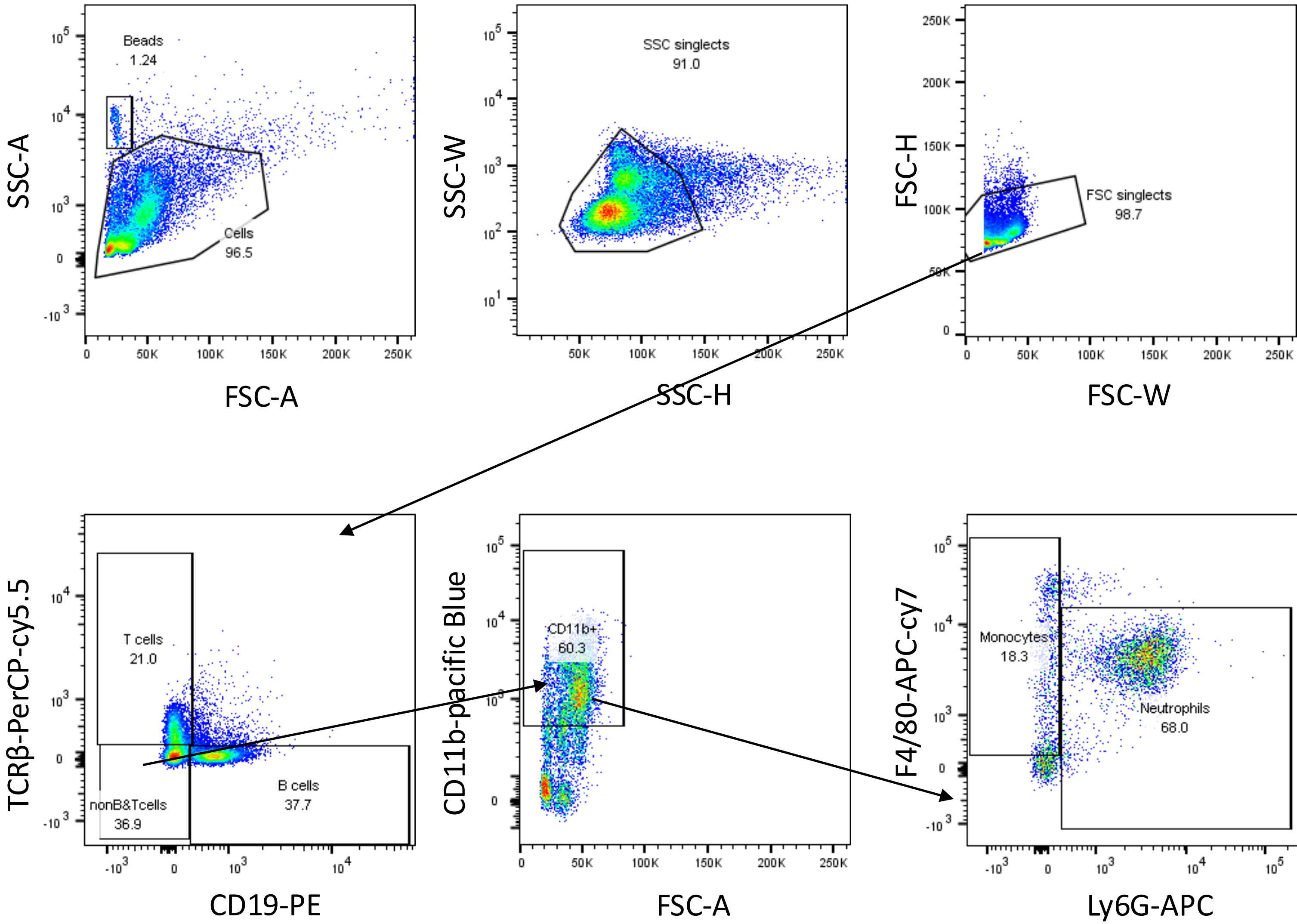

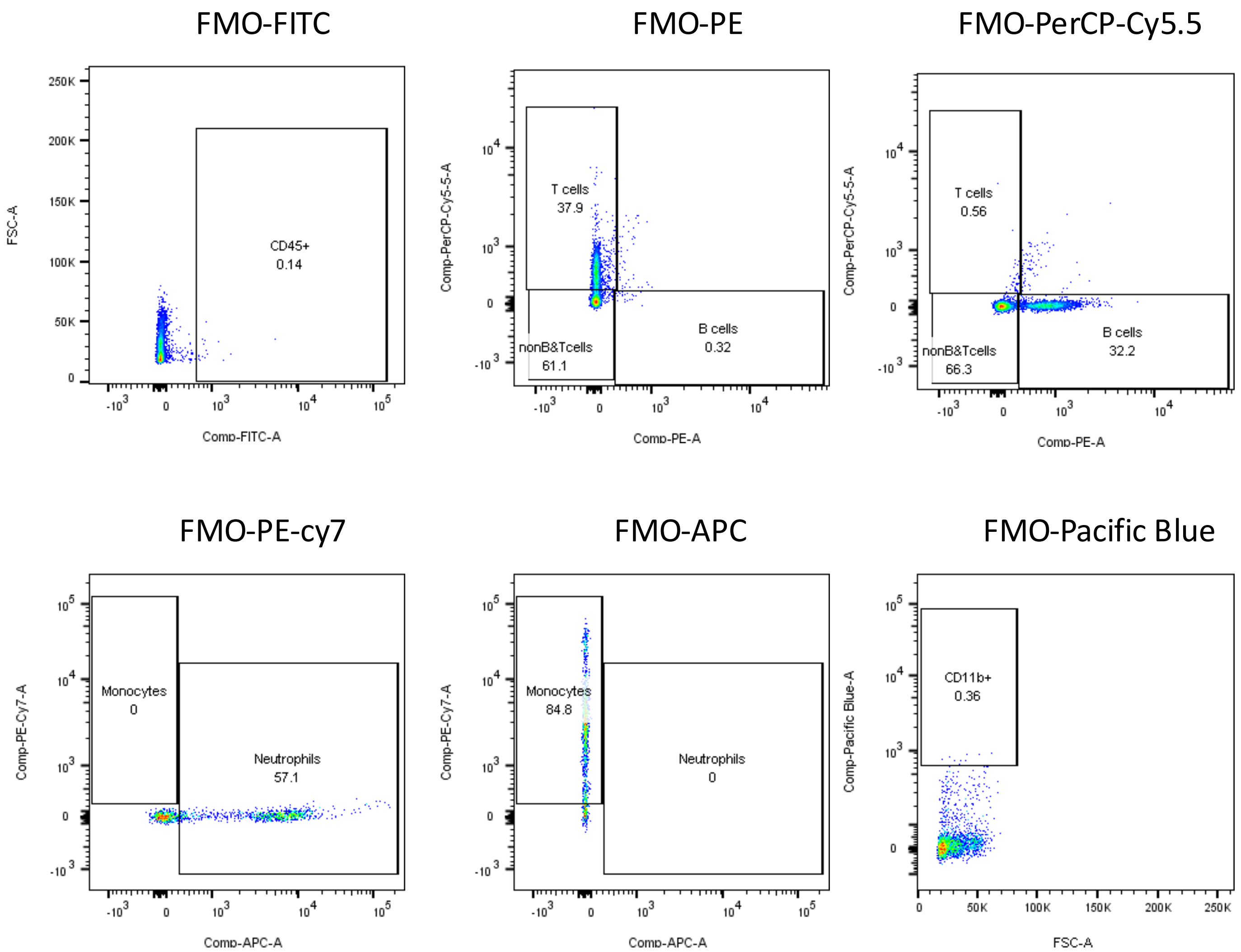
FMO-FITC
CD45+
0.14
FSC-A
Comp-FITC-A
FMO-PE
T cells
37.9
nonB&Tcells
61.1
B cells
0.32
Comp-PerCP-Cy5-5-A
Comp-PE-A
FMO-PerCP-Cy5.5
T cells
0.56
nonB&Tcells
66.3
B cells
32.2
Comp-PerCP-Cy5-5-A
Comp-PE-A
FMO-PE-cy7
Monocytes
0
Neutrophils
57.1
Comp-PE-Cy7-A
Comp-APC-A
FMO-APC
Monocytes
84.8
Neutrophils
0
Comp-PE-Cy7-A
Comp-APC-A
FMO-Pacific Blue
CD11b+
0.36
Comp-Pacific Blue-A
FSC-A

Suppl. Figure 2L

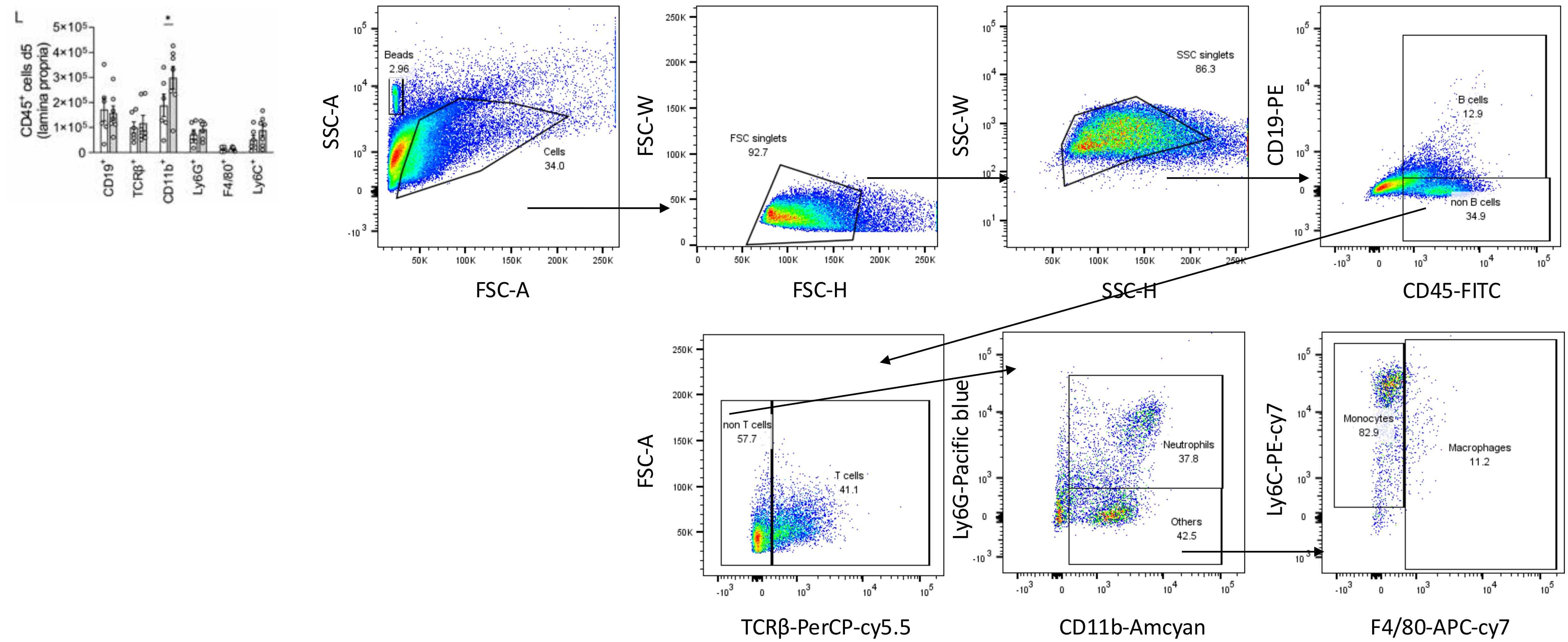

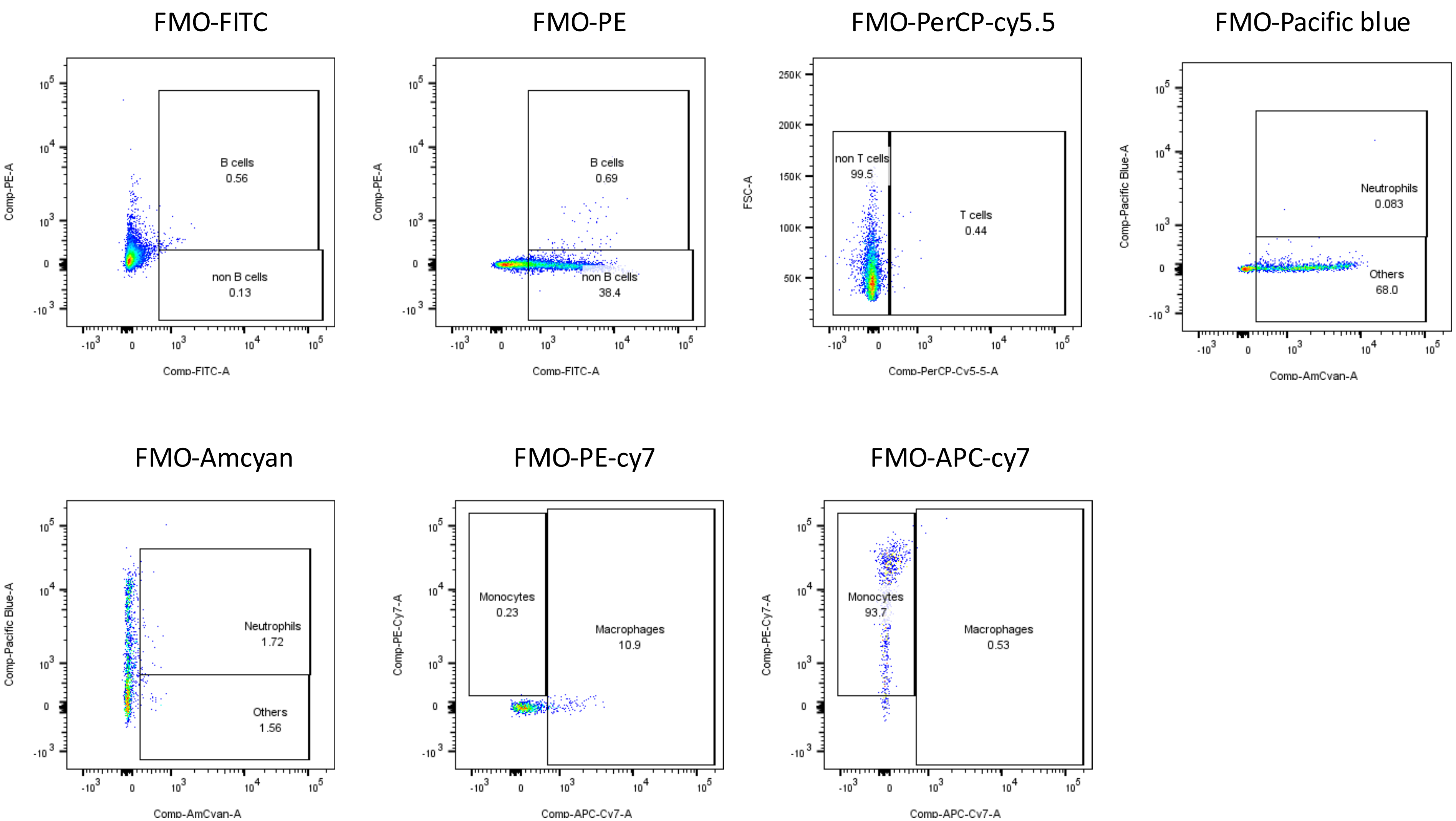
FMO-FITC
B cells
0.56
non B cells
0.13
Comp-PE-A
Comp-FITC-A
FMO-PE
B cells
0.69
non B cells
38.4
Comp-PE-A
Comp-FITC-A
FMO-PerCP-cy5.5
non T cells
99.5
T cells
0.44
FSC-A
Comp-PerCP-Cy5-5-A
FMO-Pacific blue
Neutrophils
0.083
Others
68.0
Comp-Pacific Blue-A
Comp-AmCyan-A
FMO-Amcyan
Neutrophils
1.72
Others
1.56
Comp-Pacific Blue-A
Comp-AmCyan-A
FMO-PE-cy7
Monocytes
0.23
Macrophages
10.9
Comp-PE-Cy7-A
Comp-APC-Cy7-A
FMO-APC-cy7
Monocytes
93.7
Macrophages
0.53
Comp-PE-Cy7-A
Comp-APC-Cy7-A

Suppl. Figure 3E

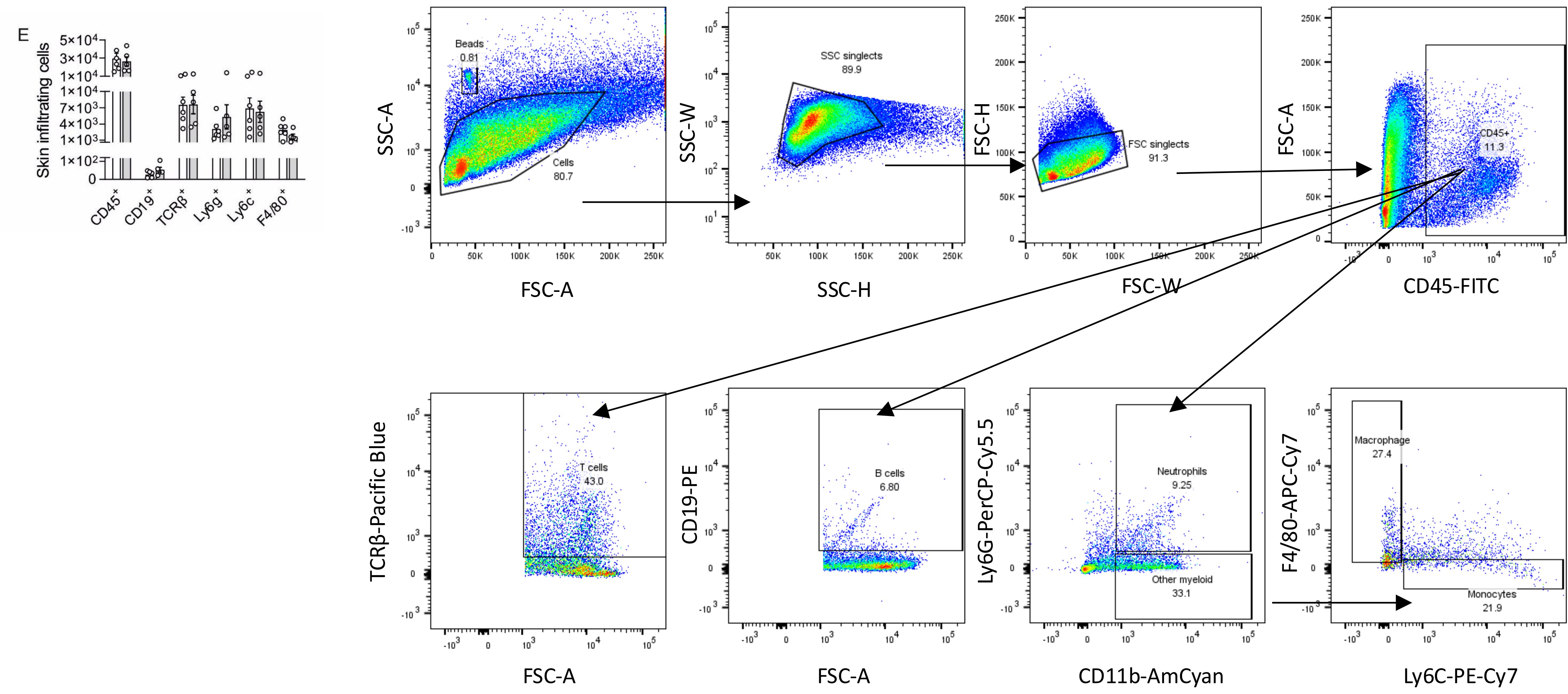

FMO-FITC

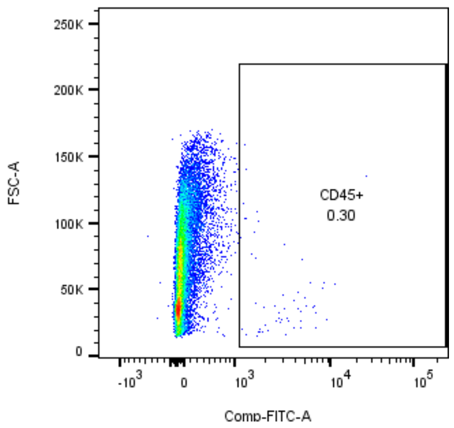

FMO-PE

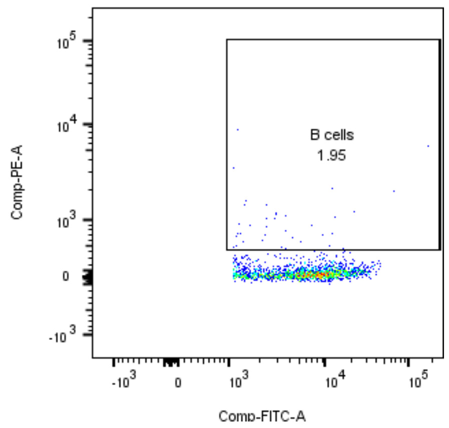

FMO-Pacific Blue

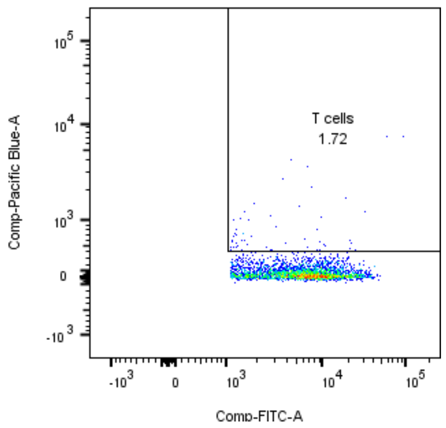

FMO-AmCyan

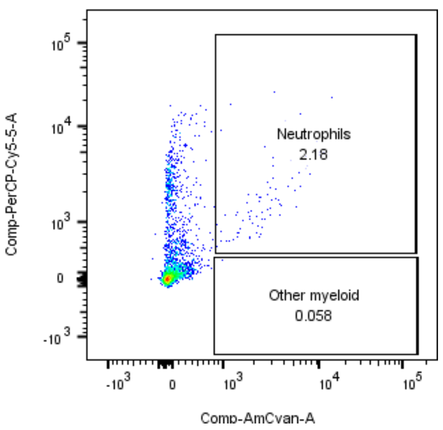

FMO-PerCP-Cy5.5

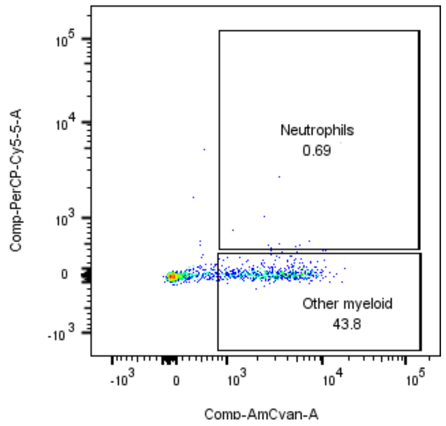

FMO-APC-Cy7

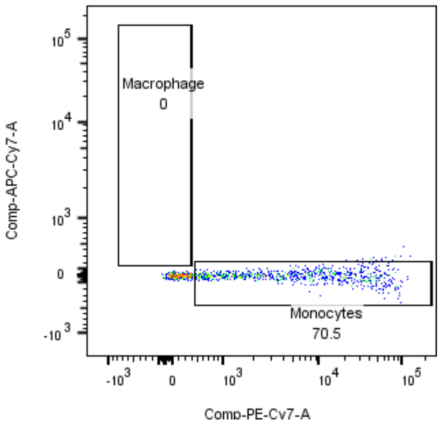

FMO-PE-Cy7

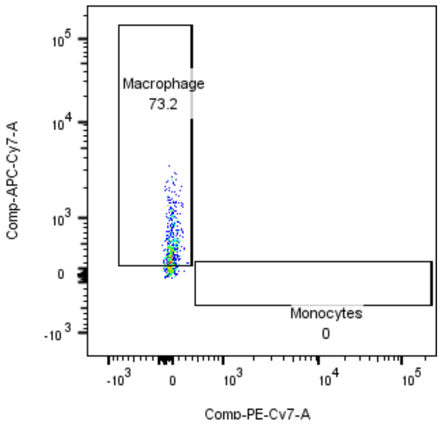

Suppl. Figure 3F

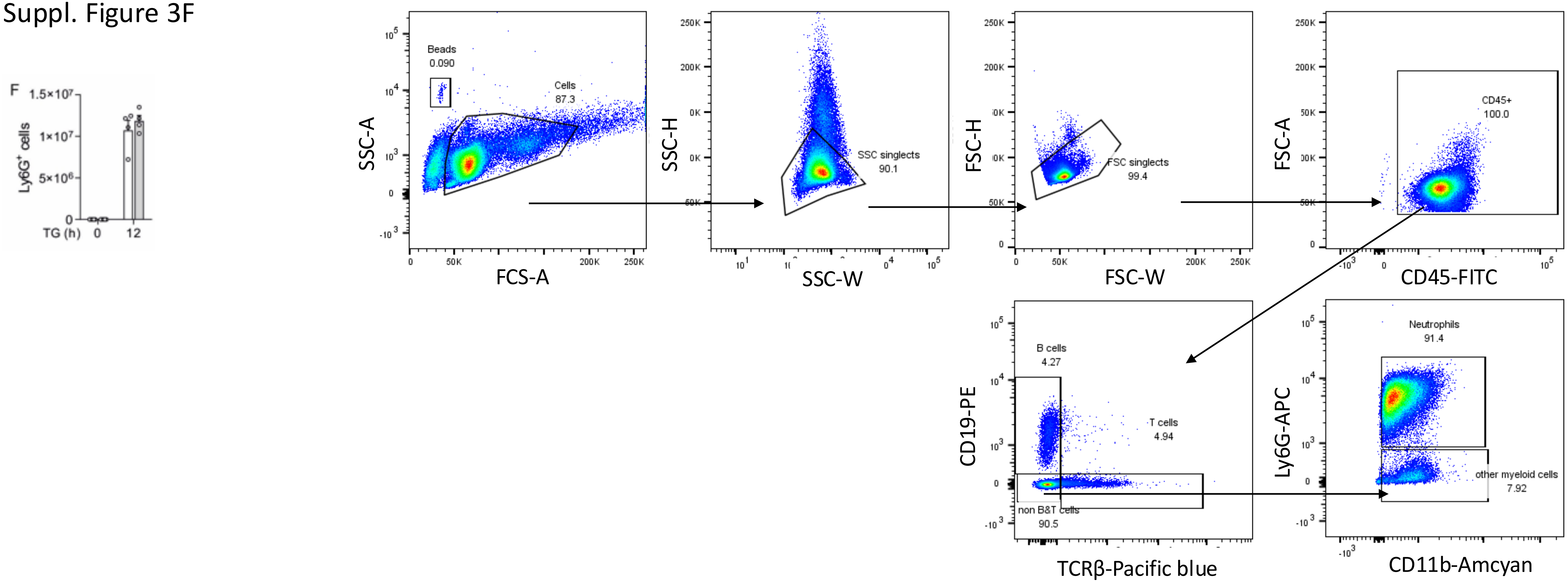

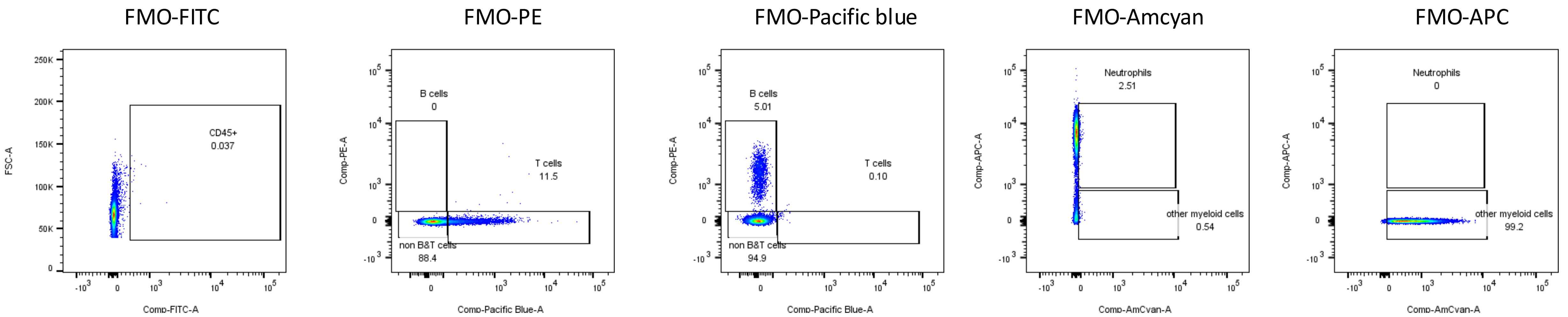
FMO-FITC
FMO-PE
FMO-Pacific blue
FMO-Amcyan
FMO-APC
FSC-A
Comp-FITC-A
CD45+
0.037
Comp-PE-A
Comp-Pacific Blue-A
B cells
0
T cells
11.5
non B&T cells
88.4
B cells
5.01
T cells
0.10
non B&T cells
94.9
Comp-APC-A
Comp-AmCyan-A
Neutrophils
2.51
other myeloid cells
0.54
Neutrophils
0
other myeloid cells
99.2

Peritoneal macrophage isolation

Before MACS

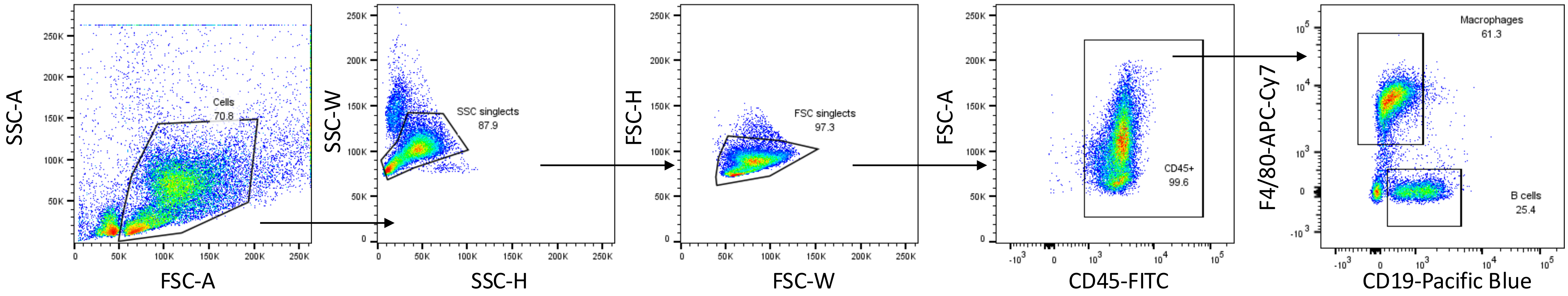

After MACS

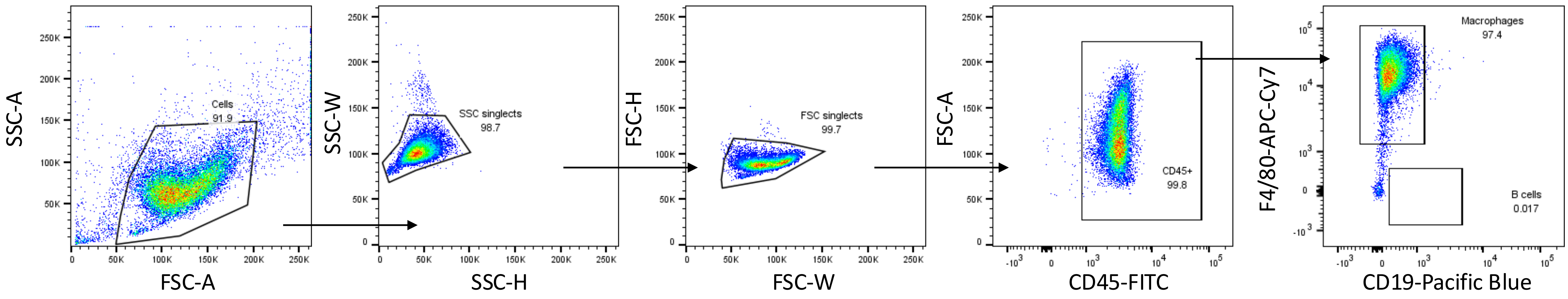

After BMDM differentiation

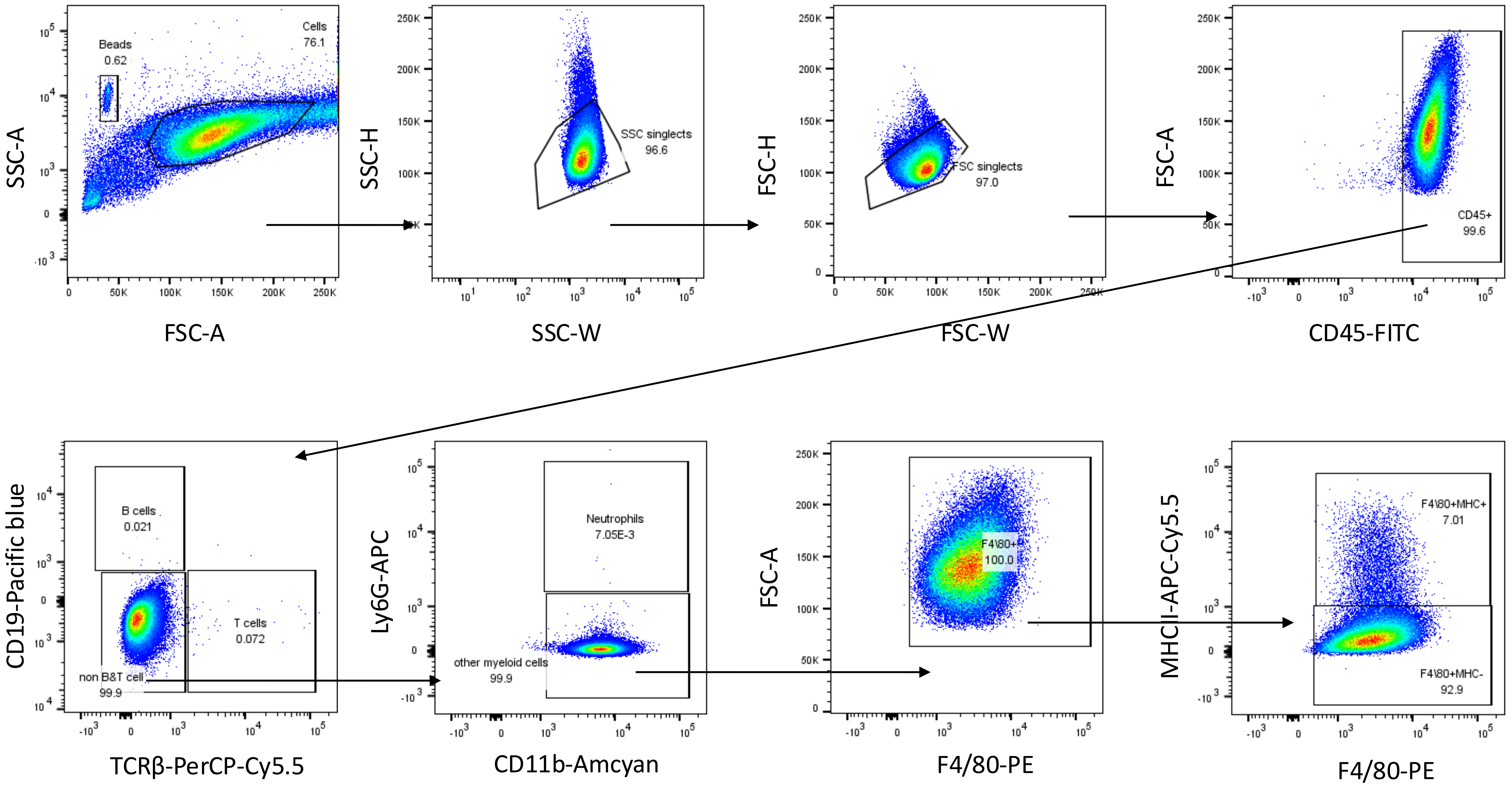

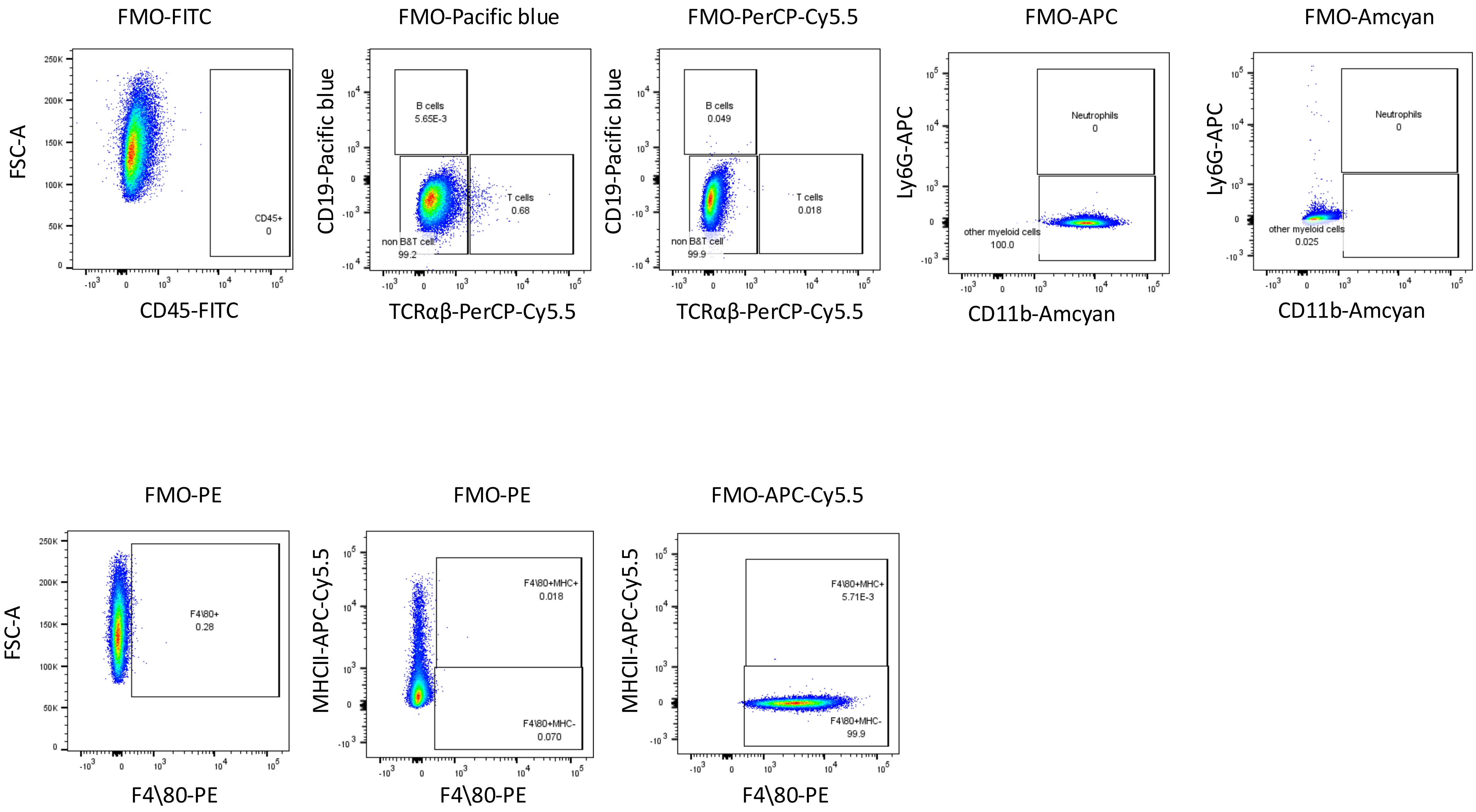
FMO-FITC
FSC-A
CD45-FITC
CD45+
0
FMO-Pacific blue
CD19-Pacific blue
TCRαβ-PerCP-Cy5.5
B cells
5.65E-3
T cells
0.68
non B&T cell
99.2
FMO-PerCP-Cy5.5
CD19-Pacific blue
TCRαβ-PerCP-Cy5.5
B cells
0.049
T cells
0.018
non B&T cell
99.9
FMO-APC
Ly6G-APC
CD11b-Amcyan
Neutrophils
0
other myeloid cells
100.0
FMO-Amcyan
Ly6G-APC
CD11b-Amcyan
Neutrophils
0
other myeloid cells
0.025
FMO-PE
FSC-A
F4\80-PE
F4\80+
0.28
FMO-PE
MHCII-APC-Cy5.5
F4\80-PE
F4\80+MHC+
0.018
F4\80+MHC-
0.070
FMO-APC-Cy5.5
MHCII-APC-Cy5.5
F4\80-PE
F4\80+MHC+
5.71E-3
F4\80+MHC-
99.9
